# *Porphyromonas gingivalis*-derived outer membrane vesicles promote vascular endothelial glycocalyx injury via the PPAD/CitH3/B3GAT1 pathway

**DOI:** 10.1186/s12951-025-04015-4

**Published:** 2026-01-09

**Authors:** Shoucheng Yin, Qihui Qiao, Zhaorong Li, Lijie Lu, Muzhou Jiang, Hanyu Gao, Ziming Ge, Chen Li, Yaping Pan, Li Lin

**Affiliations:** 1 Department of Periodontology, School and Hospital of Stomatology, China Medical University, Shenyang, Liaoning P. R. China; 2 Department of Oral and Maxillofacial Surgery, School and Hospital of Stomatology, China Medical University, Shenyang, Liaoning P. R. China; 3 Liaoning Provincial Key Laboratory of Oral Disease, Shenyang, Liaoning P. R. China

**Keywords:** Cardiovascular disease, Glycocalyx, Periodontitis, Clinical attachment loss, Periodontal probing depth, Bleeding on probing

## Abstract

**Background:**

The glycocalyx serves as the skeletal structure of the outer layer of endothelial cells and regulates the function of endothelial cells. *Porphyromonas gingivalis* (*P. gingivalis*) outer membrane vesicles (OMVs) exhibit the fundamental biological traits of bacteria, such as inducing inflammatory responses, damaging host cells, and delivering virulence factors to distal tissues like the cardiovascular system. This study aimed to investigate the role of *P. gingivalis* OMVs in vascular endothelial glycocalyx injury.

**Methods:**

In this clinical study, serum levels of syndecan-1 (SDC1) and heparan sulfate (HS), biomarkers of endothelial glycocalyx injury, were measured and compared between patients with stage III-IV periodontitis and those with stage I-II or no periodontitis. Then, glycocalyx injury was detected using transmission electron microscopy, immunofluorescence and western blotting after vascular endothelial cells were stimulated and C57BL/6J mice were administered with *P. gingivalis* OMVs via tail vein injection. Transcriptomic high-throughput sequencing analysis and in vitro and in vivo rescue experiments were conducted to determine the key mechanism in glycocalyx injury. Experiments were conducted using OMVs, ^PPAD−OE^OMVs, and ^ΔPPAD^OMVs to identify the special virulence factors in OMVs.

**Results:**

This study revealed that serum levels of SDC1 and HS were significantly higher in patients with stage III-IV periodontitis (*P* < 0.05). A marked reduction in both the fluorescence intensity of the glycocalyx and the expression levels of its key components was observed in the OMVs group compared with control group (*P* < 0.05). We identified the key differentially expressed gene *B3GAT1* using high-throughput sequencing. Subsequent rescue experiments both in vitro and in vivo demonstrated that overexpression of B3GAT1 effectively restored glycocalyx integrity following injury (*P* < 0.05). Notably, *Porphyromonas gingivalis* peptidylarginine deiminase (PPAD) was found to promote endovascular glycocalyx injury by citrullinating histone H3, thereby decreasing the expression of B3GAT1 (*P* < 0.05).

**Conclusions:**

Our experiments demonstrated that biomarkers of endothelial glycocalyx injury were significantly higher in patients with stage III-IV periodontitis and PPAD could enter the cell nucleus, playing a vital role in vascular endothelial glycocalyx injury through the CitH3/B3GAT1 pathway.

**Graphical abstract:**

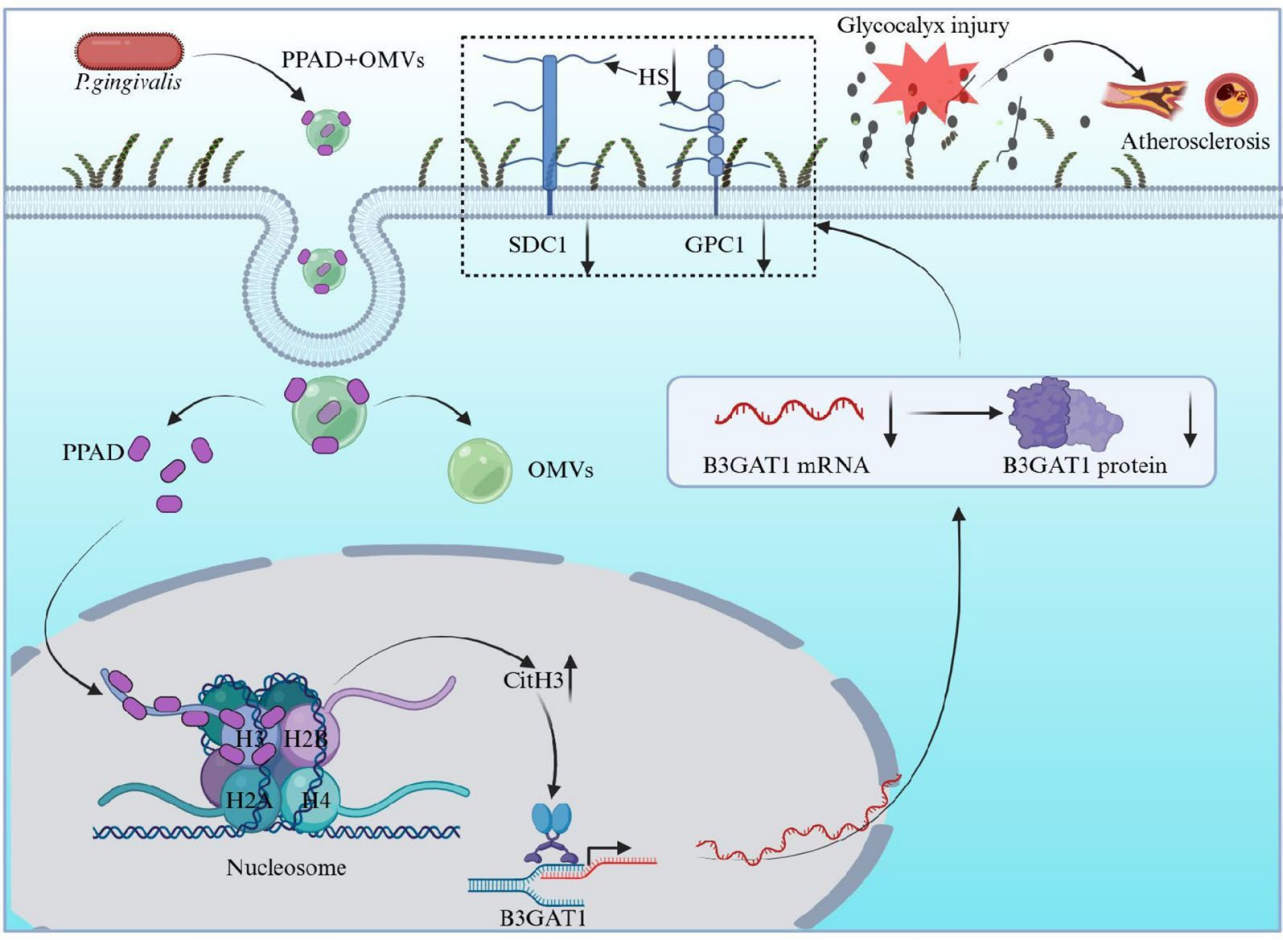

**Supplementary Information:**

The online version contains supplementary material available at 10.1186/s12951-025-04015-4.

## Introduction

Periodontitis is one of the most common chronic inflammatory non-communicable diseases in humans, affecting approximately half of the adult population worldwide. Further, the incidence of severe periodontitis has shown a slight increase in recent years [1, 2]. It is a destructive, inflammatory disease, initiated by periodontal pathogenic bacteria that damages periodontal supporting tissues [3]. Local stimulation of periodontal pathogens can induce chronic inflammation of periodontal tissues, increase the level of inflammatory cytokines in the circulatory system, and mediate systemic inflammation, thereby affecting the occurrence and development of systemic diseases [4]. Moreover, periodontal pathogens and their products may enter the circulatory system through the damaged periodontal epithelium, colonize the heart and other important organs through blood circulation, and also contribute to the occurrence and development of systemic diseases [4]. *Porphyromonas gingivalis* (*P. gingivalis*), a known periodontal pathogen, can release stable outer membrane vesicles (OMVs), which are internalized into host tissues and activate proinflammatory pathways in host cells [5]. *P. gingivalis* OMVs not only bear the main components of bacterial outer membranes but also serve as carriers of various virulence factors produced by bacteria. Due to the protection provided by the vesicle membrane structure, high concentrations of pathogenic factors can evade degradation and achieve long-distance transmission. This characteristic increases the virulence of *P. gingivalis* compared to that of other bacteria [6, 7]. Recently, studies on the relationship between *P. gingivalis* OMVs and cardiovascular disease (CVD) have been gaining increasing attention.

The glycocalyx, a multi-villous structure on the lateral surface of vascular endothelial cells, serves as the skeletal structure of the outer layer of endothelial cells and maintains the homeostasis of the vascular system, regulating the function of endothelial cells [8, 9]. Glycocalyx is a gelatinous substance that binds to the luminal surface of vascular endothelial cells, and is mainly composed of proteoglycans (PGs), glycosaminoglycans (GAGs), membrane glycoproteins, and plasma proteins, of which PGs and GAGs are its main components [10, 11]. PGs primarily consist of Syndecan-1 (SDC1) and Core Protein (Glypican), which are prone to binding with GAG chains. GAGs encompass heparan sulfate (HS), hyaluronic acid (HA), chondroitin sulfate, and dermatan sulfate [9, 11]. As a vascular endothelium barrier, glycocalyx plays an important role in regulating vascular permeability, maintaining vascular tension, preventing thrombosis, mediating shear induced nitric oxide (NO) production, and regulating leukocyte adhesion [9, 12]. Glycocalyx injury can cause structural changes in endothelial cells, increase their permeability, promote lipid deposition in blood vessel walls, and trigger the release of inflammatory factors. These changes induce monocyte adhesion, macrophage infiltration, and foam cell formation as well as accelerate the progression of CVDs [9, 13]. HS and SDC1, which are typically shed into the bloodstream, can be used as biomarkers of glycocalyx injury to monitor the severity of vascular endothelial injury [14, 15].

Current studies have shown that *P. gingivalis* OMVs could increase the permeability of human vascular endothelial cells (VECs), resulting in increased tissue exudate and tissue edema. It can also promote the occurrence of atherosclerosis (AS) by inhibiting the effect of NO, resulting in increased vasoconstriction and apoptosis of endothelial cells [16, 17].Other studies have shown that *P. gingivalis* OMVs are involved in bacterial adhesion, biofilm formation, invasion, and damage to host cells, thereby altering the regulation of host immune responses [18, 19]. Therefore, this study aimed to examine the role of *P. gingivalis* OMVs in vascular endothelial glycocalyx injury and elucidate the specific associated mechanism.

## Materials and methods

### Clinical experiments

The study participants were periodontitis patients admitted to the Department of Periodontology, Hospital of Stomatology, China Medical University from January 2023 to September 2024, as well as healthy individuals with no periodontal disease.

The diagnostic criteria for periodontitis was based on to the new classification of periodontal and peri-implant diseases and conditions [20], defined as the detection of two non-adjacent teeth with adjacent clinical attachment loss (CAL) ≥ 1 mm or buccal or oral CAL ≥ 3 mm, and probing pocket depth (PPD) ≥ 3 mm in ≥ 2 teeth at the same time.

Periodontitis stages [21] were defined as (1) stage I periodontitis: maximum adjacent CAL 1 ~ 2 mm, PPD ≤ 4 mm, and no missing tooth due to periodontitis; (2) stage II periodontitis: maximum adjacent CAL 3 ~ 4 mm, PPD ≤ 5 mm, and no missing tooth due to periodontitis; (3) stage III periodontitis: maximum adjacent CAL ≥ 5 mm, PPD ≥ 6 mm, and missing tooth due to periodontitis ≤ 4, furcation II or III; (4) stage IV periodontitis: maximum adjacent CAL ≥ 5 mm, PPD ≥ 6 mm, missing tooth due to periodontitis ≥ 5, secondary occlusal trauma, and remaining teeth < 20.

Inclusion criteria for the healthy individuals without periodontal disease included: (1) PPD < 3 mm; (2) bleeding on probing (BOP) < 10%; (3) CAL = 0; (4) no alveolar bone loss, and exclusion criteria included (1) Age < 20 years; (2) presence of systemic diseases such as CVD and diabetes without effective control; (3) periodontal treatment within 3 months, (4) used antibiotics, sulodexide, or any other drugs within 3 months that may affect the experimental results; (5) female in menstrual period, pregnancy, or lactation.

The serum HS and SDC1 levels were measured using their respective enzyme-linked immunosorbent assay kits (CEA161Ge and SEB966Hu, Cloud-Clone Corp., China).

### Statistical analysis for the clinical experiments

Data were analyzed using SPSS 26.0. For continuous variables, the Kolmogorov-Smirnov test was used to test for normality, and the data was deemed not to follow a normal distribution when was *P* < 0.05. Data following a normal distribution were summarized as mean ± standard deviation, and independent sample *t*-tests were used for inter-group comparisons. Data with skewed distribution were summarized as median (interquartile range, P_25_, P_75_), and two independent sample non-parametric tests (Mann-Whitney test) were used for inter-group comparisons. For categorical variables, the chi-square test was employed to compare the differences among different groups. When the total sample size was ≥ 40 and the theoretical frequency T was ≥ 5, the Pearson chi-square test was utilized. If 1 ≤ T < 5 and *n* ≥ 40, the continuity-corrected chi-square test was applied. If T < 1 or *n* < 40, Fisher’s test was used. Due to the non-normal distribution of continuous variables, Spearman correlation analysis was employed to examine the correlation of HS andSDC1with various parameters including PPD, CAL, and BOP. A correlation heat map and scatter plot were used to illustrate the relationship between the two parameters, and statistical graphs were created using R Studio. After dichotomizing HS and SDC1 according to the median, the correlation between SDC1, HS, and periodontitis was analyzed by logistic regression using multivariate adjustment. A receiver operating characteristic (ROC) curve and area under the curve (AUC) were employed to assess the predictive power of organic SDC1 and HS for periodontitis. All tests were conducted on a bilateral basis, with significance threshold set at *P* < 0.05.

### Bacterial culture

*P. gingivalis* W83 was cultured on brain heart infusion (BHI) medium (04–016, Aobox, China) supplemented with 0.005‰ hemin (H1393, Jintai Hongda Biotechnology, China), 0.1% Phylloquinone K1 (JANGSU HUAYANG PHARMACEUTICAL, China), and 5% defibrinated sheep blood (1001339-1, Hoprbio, China) in an anaerobic atmosphere of 10% CO2, 80% N2, and 10% H2 at 37 °C. *P. gingivalis* ΔPG1424 was cultured with additional 0.5‰ erythromycin (114-07-8, Solarbio, China) and *P. gingivalis* PPAD-OE was cultured with additional 1‰ ampicillin sodium (A1170, Solarbio, China). Three strains of *P. gingivalis* were provided by the Department of Oral Biology, School and Hospital of Stomatology, China Medical University, Shenyang, China.

### Isolatoion and purification of *P. gingivalis* OMVs

*P. gingivalis* OMVs were isolated using an established protocol [16]. Briefly, after *P. gingivalis* was cultured anaerobically in 200 mL BHI medium at 37 °C for 2 days, the freshly grown bacterial cultures (OD_600_ = 1, equivalent to 9 × 10^9^ colony-forming units) were centrifuged (8,000 g, 4 °C, 20 min) and the pellet collected. The supernatant was filtered (0.22-µm filter) and further centrifuged for 1 h at 100,000 g and 4 °C. The resulting OMVs pellet was washed once with PBS, ultracentrifuged again, resuspended in PBS, stored at −80 °C for future use, and later characterized using nanoparticle tracking analysis (NTA), and transmission electron microscopy (TEM).

### Cell culture

Human umbilical vein endothelial cells (EA. hy926, Cat NO.: CL-0272, Procell, China) and human acute monocytic leukemia cells (THP-1, Cat NO.: CL-0233, Procell, China) were respectively cultured in DMEM (MA0212, MeilunBio, China) and RPMI-1640 media (MA0215, MeilunBio, China) containing 10% fetal bovine serum (164210, Procell, China) and were maintained in a constant temperature incubator of 37 °C and 5% CO2. Cells were passaged when the confluence reached approximately 80%. After digestion and neutralization, the cell suspension was transferred to a centrifuge tube, centrifuged at 900 g/min for 4 min, the supernatant was discarded, and the cells were resuspended in complete medium, and subsequently subcultured at a ratio of 1:3. Cell growth was observed, and the cells in the logarithmic growth phase were selected for subsequent experiments.

### Cell counting kit-8 (CCK-8) assay

EA. Hy926 cells were seeded into 96-well plates for 24 h, and different concentrations of *P. gingivalis* OMVs (0, 1,3, 5, 8, 10, 12, and 15 µg/mL) were used to stimulate the cells. At 6 h, 12 h, 24 h, 48 h, and 72 h after treatment with *P. gingivalis* OMVs, 10% CCK-8 solution (KTA1020, Abbkine, China) was added to the wells and the absorbance was measured at 450 nm after 1 h.

### Outer membrane vesicles labeling and internalization

An amount of 5 µM DID (C1039, Beyotime, China) was incubated with *P. gingivalis* OMVs for 30 min to label the *P. gingivalis* OMVs in accordance with a previous study. Then, DID-labeled *P. gingivalis* OMVs were stimulated with EA. hy926 cells and co-cultured for 2 h. The cells were fixed with 4% paraformaldehyde (PFA) and sealed with 5% BSA for 1 h. Cells were then incubated with FITC-Phalloidin (CA1620, Solarbio, China) overnight at 4 °C to label the cytoskeleton and stained with DAPI for 10 min to visualize the cell nucleus. A confocal laser scanning microscope (CLSM) (NIKON ECLIPSE Ti2, Japan) was used to obtained fluorescence images of EA. Hy926 cells. In vivo, DID-labeled *P. gingivalis* OMVs were injected into C57BL/6J mice through the tail vein, and the samples were collected 2 h later. The fluorescence intensity of the hearts and aortas of mice was measured using the in vivo imaging system (IVIS) (PerkinElmer, USA).

### Grouping and experimental design in vitro

First, EA. Hy926 cells were seeded into six-well plates for 24 h, and different concentrations of *P. gingivalis* OMVs (0, 1, 2, 5, 8, and 10 µg/mL) were used to stimulate the cells to determine the optimal concentration necessary to damage the glycocalyx. The optimal time point of glycocalyx damage was examined at 0 h, 6 h, 12 h, and 24 h after treatment with *P. gingivalis* OMVs. After identifying the appropriate siRNA, EA. hy926 cells were divided into PBS, A1, OE, OMVs, and OE + OMVs groups and stimulated with *P. gingivalis* OMVs (5 µg/mL) for 12 h. Finally, EA. hy926 cells were divided into the PBS, ^ΔPPAD^OMVs, and OMVs groups, and the PBS and *P. gingivalis* ^ΔPPAD^OMVs (5 µg/mL) groups were stimulated for 12 h.

The experiment of monocytes adhesion to endothelial cells was conducted in accordance with the protocol of the Calcein AM Cell Viability Assay kit (C2013L, Beyotime, China) 12 h after EA. hy926 cells were stimulated *P. gingivalis* OMVs.

### Grouping and experimental design in vivo

Eight-week-old male C57B/6J mice were obtained from BEIJING HFK BIOSCIENCE Co., LTD, China. Three stage experiments were conducted successively. In the first stage, mice were first divided into PBS and OMVs group, with 10 mice in each group. In the second stage, mice were divided into PBS (10), OMVs (10), OE_AAV_+OMVs (10), and OE_AAV−NC_ (2) groups. In the third stage, mice were divided into PBS, OMVs, and ^ΔPPAD^OMVs groups, with 10 mice in each group. In the OMVs and OE_AAV_+OMVs groups, each mouse was injected with *P. gingivalis* OMVs equivalent quantities of 100 µg protein in 0.2 ml PBS once every other day through the tail vein for one month. In the ^ΔPPAD^OMVs group, each mouse was injected with *P. gingivalis* ^ΔPPAD^OMVs similar to the above. In the PBS group, each mouse was injected with 0.2 mL PBS. In particular, mice in the OE_AVV_+OMVs group were injected with 5e + 11v.g. adeno-associate virus (AAV), which could overexpress B3GAT1 through the tail vein before the start of the experiment, and samples were taken one week later to verify the successful transfection of AVV. Each mouse received 5e + 11v.g. AAV-NC in the OE_AAV−NC_ group. The animal experiments were conducted according to protocols of previous successful experiments [22, 23].

### Reverse transcriptase-quantitative polymerase chain reaction (RT-qPCR)

Total RNA was extracted from EA. hy926 cells using the Trizol reagent (Invitrogen, Waltham, MA, USA) in accordance with the manufacturer’s instructions. Then, 1 µg of RNA was reverse transcribed to complementary DNA (cDNA) using a PrimeScript™ RT reagent kit with gDNA Eraser (RR047A, TaKaRa, Kusatsu, Japan) and quantified using the SYBR Green reagent (RR820A, TaKaRa, Kusatsu, Japan) in a CFX96 real-time PCR detection system (Bio-Rad, Hercules, CA, USA). The 2^−ΔΔCt^ method was used to calculate the relative expression. The gene-specific primers for the RT-qPCR were shown in Table S2.

### Immunofluorescence (IF) staining

Cell sections were fixed using 4% PFA and sealed with 5% BSA for 1 h. Tissue sections were immersed in xylene for dewaxing and graded alcohol for rehydration, followed by heatinduced epitope retrieval. Then, the sections were treated with 3% H_2_O_2_ for 30 min and then 5% goat serum for 1 h. The sections were then treated with anti-FITC-WGA (wheat germ agglutinin) (GTX01502, GeneTex,1: 800, no secondary antibody necessary), anti-HS (ab2501, Abcam, 1:1000), anti-Histone H3 (ab1971, Abcam, 1:1000), anti-His-Tag (66005-1-Ig, Proteintech, 1:800), and anti-FLAG (#2368, Cell Signaling, 1:200) at 4 °C for 12 h. Next, the specimens were incubated with fluorescein-conjugated anti-rat, anti-rabbit and anti-mouse IgG secondary antibody (Alexa Fluor 488, ab150157, Abcam, 1:200; Coralite Plus 594, RGAM004, Proteintech, 1: 800; Coralite Plus 488, RGAR002, Proteintech,1: 800 and Coralite Plus 594, RGAR004, 1: 800) at room temperature for 1 h. Nuclei were stained with DAPI (P0131, Beyotime, China). Fluorescence images of the sections were obtained using a microscope slide scanner. Quantitative analysis of the fluorescence intensity was performed using Image J software.

### Western blotting (WB)

Equal amounts of protein were isolated by SDS-PAGE and transferred to PVDF membranes, followed by blocking for 30 min at room temperature. The proteins were incubated with anti-HS (ab2501, Abcam, 1:2000); anti-SDC1 (A4174, Abclonal, 1:2000); anti-GPC1 (A13019, ABclonal, 1:2000); anti-B3GAT1 (A9871, ABclonal, 1:2000); anti-H3 (ab1791, Abcam, 1:3000), anti-CitH3 (ab281584, Abcam, 1:1000), anti-RL2 (ab2739 and ab93858, Abcam, 1:1000), anti-His-Tag (AE104, Abclonal, 1:5000, no secondary antibody needed), anti-FLAG (#2368, Cell Signaling, 1:1000) and anti-β-Actin (HRP-66009, Proteintech, 1: 5000, no secondary antibody needed) at 4 °C overnight. The membrane was subsequently incubated with HRP-conjugated secondary antibody (AS003, AS014 and AS028, Abclonal, 1:5000) for 1 h at room temperature. Bands were visualized using ECL detection reagents. For quantitative analysis, the results were analyzed using Image J software, normalized by β-Actin.

### Nucleoplasmic separation and CoIP

EA. hy926 cells were stimulated with *P. gingivalis* ^PPAD−OE^OMVs. Nuclear and plasma proteins were obtained using the protocol of the MeiLun Nuclear and Cytoplasmic Protein Extraction kit (MA0211, MeilunBio, China). The co-immunoprecipitation was conducted in accordance with the protocol of the rProtein A/G Magnetic IP/Co-IP kit (abs9649, Absin, China). The co-immunoprecipitation protein was subsequently analyzed by liquid chromatography tandem mass spectrometry (LC-MS/MS) to identify the protein that binds to PPAD-His-Tag.

### Prediction of transcription regulatory factors and docking of computer simulated molecules

ChEA3 database (https://maayanlab.cloud/chea3/) was used to predict *B3GAT1*-related transcription adjustment factor. Molecular docking was used to verify the binding activity between protein-protein or protein-small molecule interactions. The structures of PPAD, Histone H3 and Kv channel interacting protein 3 (KCNIP3) were obtained from AlphaFold, and the structure of B3GAT1 was sourced from RNAComposer. The interactions were performed using H-dock. The best docking model was selected based on the score and active site for visualization. The interaction sites of the docking model were displayed in Discovery Studio.

### Statistical analysis

The results of at least three independent experiments (*n* ≥ 3) were presented as mean ± SD and graphs were drawn using GraphPad Prism. Statistical differences were analyzed using two-tailed Student’s t test to evaluate the variability between the two groups, and one-way analysis of variance (ANOVA) was used to evaluate the variability between multiple groups. Fisher’s protected least significant differences was also used, and the threshold of significance levels was set at 0.05 in each analysis.

## Results

### Serum levels of SDC1 and HS were significantly increased in patients with stage III-IV periodontitis

**Fig. 1:**
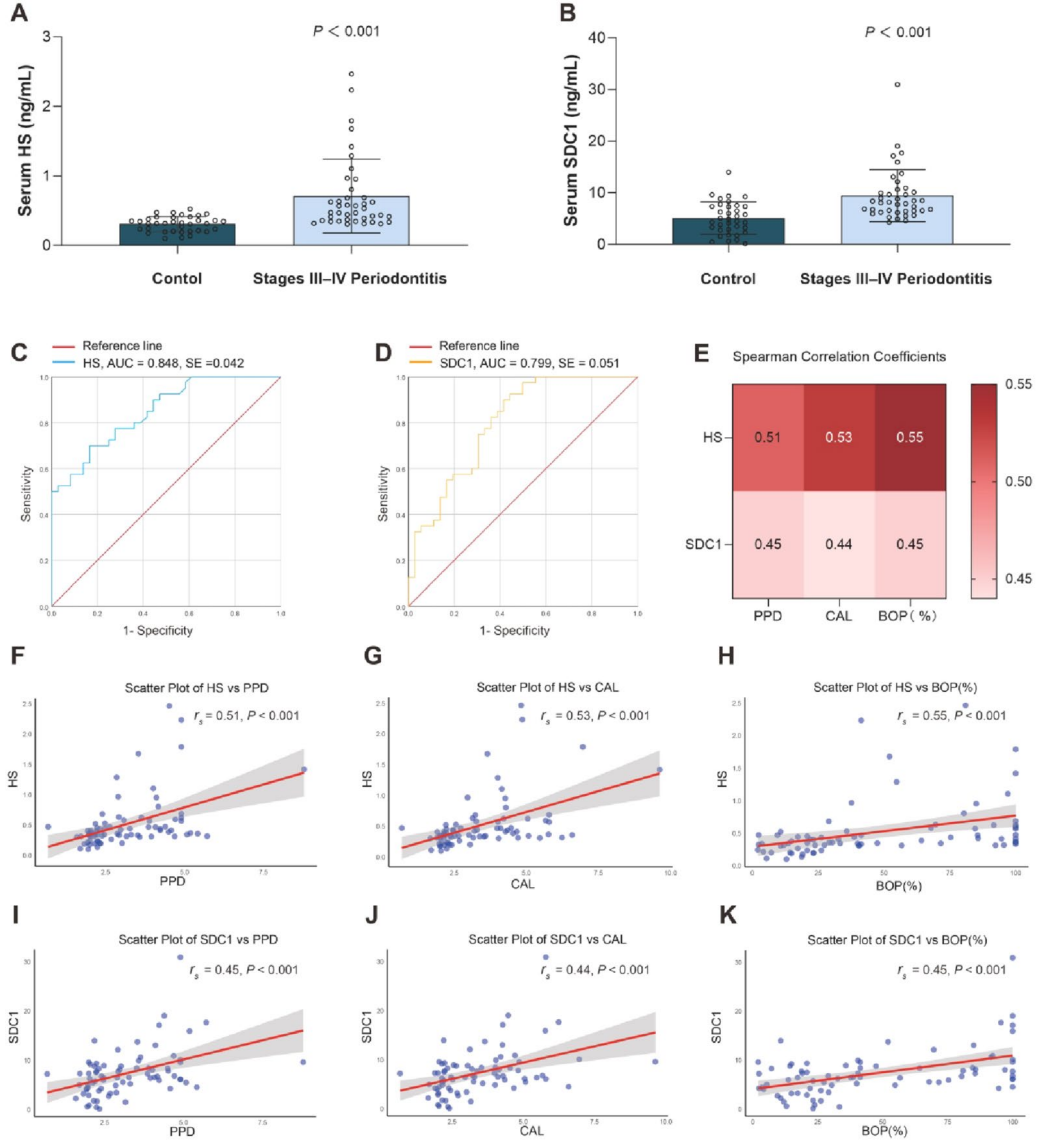
Clinical study: Comparisons of clinical parameters in patients with stages III-IV periodontitis and participants with no/stages I-II periodontitis (**A**) Serum HS concentration; (**B**) Serum SDC1 concentration; (**C**) ROC curve analysis of HS for predicting periodontitis status; (**D**) ROC curve analysis of SDC1 for predicting periodontitis status; (**E**) Heat map of correlation of PPD, CAL and BOP with HS and SDC1; (**F**-**K**) Scatter plots of correlations between PPD, CAL and BOP and HS and SDC1, respectively. *Abbreviation* AUC, area under the curve; BOP, bleeding on probing; CAL, clinical attachment loss; HS, heparan sulfate; PPD, periodontal probing depth; ROC, receiver operating characteristic; SDC1, syndecan-1

A total of 36 participants with no/stage I-II periodontitis and 40 participants with stage III-IV periodontitis were enrolled in this study. The incidence of stage III-IV periodontitis was 52.63%, and patients with stage III-IV periodontitis were more likely to be older, current smokers, and moderate drinkers (*P* < 0.05). Participants with stage III-IV periodontitis were more likely to have greater numbers of missing teeth, probing PPD, CAL, BOP, HS, and SDC1 (*P* < 0.001). The differences in serum HS and SDC1 between the two groups were shown in Fig. 1A, B. The details of the study characteristics were presented in Table 1.

**Table 1:** Clinical characteristics of the participants according to their periodontal diagnosis

Characteristics	Periodontal status		*P* value
	NO/Stages I–II Periodontitis	Stages III–IV Periodontitis	
No. (%)	36 (47.37)	40 (52.63)	-
Age (years)	30.00 (24.00, 34.75)	48.00 (39.50, 55.00)	< 0.001
Sex, n (%)			0.118
female	19 (52.78)	14 (35.00)	
male	17 (47.22)	26 (65.00)	
Smoking status, n (%)			0.028
Never smoker	33 (91.67)	27 (67.50)	
Former smoker	0 (0.00)	3 (7.50)	
Current smoker	3 (8.33)	10 (25.00)	
Drinking status, n (%)			< 0.001
Non-drinker	36 (100.0)	25 (62.50)	
Light drinker	0 (0.00)	10 (25.00)	
Moderate drinker	0 (0.00)	5 (12.50)	
Hypertension status, n (%)			0.108
No	34 (94.44)	33 (82.50)	
Yes	2 (5.56)	7 (17.50)	
Diabetes status, n (%)			0.174
No	36 (100.0)	38 (95.00)	
Yes	0 (0.00)	2 (5.00)	
Missing teeth, n	0.00 (0.00, 0.00)	1.00 (0.00, 3.00)	< 0.001
PPD, mm	2.17 (1.97, 2.34)	4.09 (3.28, 4.85)	< 0.001
CAL, mm	2.19 (2.01, 2.41)	4.28 (3.54, 5.07)	< 0.001
BOP (%)	18.01 (10.71, 23.66)	82.76 (48.60, 100.00)	< 0.001
HS, ng/mL	0.32 (0.21, 0.40)	0.50 (0.36, 0.78)	< 0.001
SDC1, ng/mL	4.71 (2.79, 7.35)	8.11 (6.44, 10.51)	< 0.001

PPD, periodontal probing depth; CAL, clinical attachment loss; BOP, bleeding on probing; HS, heparan sulfate; SDC1, syndecan-1

HS and SDC1 were classified into two categories based on the median concentration (median of HS: 0.3837 ng/mL; median of SDC1: 6.6562 ng/mL). In the analysis without adjustment for covariates, stage III-IV periodontitis was significantly associated with HS (Model 0; HR, 7.91; CI, 2.84–22.05; *P* < 0.001) and SDC1 (Model 0; HR, 4.22; CI, 1.61–11.04; *P* = 0.003). Consistently, after multivariate adjustment, higher periodontitis status remained significantly associated with an increased risk of HS (Model 1; HR, 8.91; CI, 1.91–41.49; Model 2; HR, 8.72; CI, 1.21–63.05; Model 3; HR, 9.50; CI, 1.19–75.98; *P* < 0.05). However, higher periodontitis status was significantly associated with an increased risk of SDC1 only for Model 1 (HR, 5.71; CI, 1.29–25.34; *P* = 0.022) (Table S1).

Using periodontitis as an independent variable and serum HS and SDC1 levels as the dependent variables, ROC curve analysis showed that the AUC predicted by HS was 0.848 (95% CI: 0.766–0.931, *P* < 0.001) (Fig. 1C), and the AUC predicted by serum SDC1 was 0.799 (95% CI: 0.700–0.899.700.899, *P* < 0.001) (Fig. 1D). After Spearman correlation analysis, the correlation coefficients of the periodontal status indices PPD, CAL, and BOP with serum HS levels were 0.51, 0.53, and 0.55, and SDC1 levels were 0.45, 0.44, and 0.45, respectively. The correlation heat map and scatter plot are shown in Fig. 1E-K.

### ***P. gingivalis*** OMVs promoted glycocalyx injury in vascular endothelial cells

**Fig. 2:**
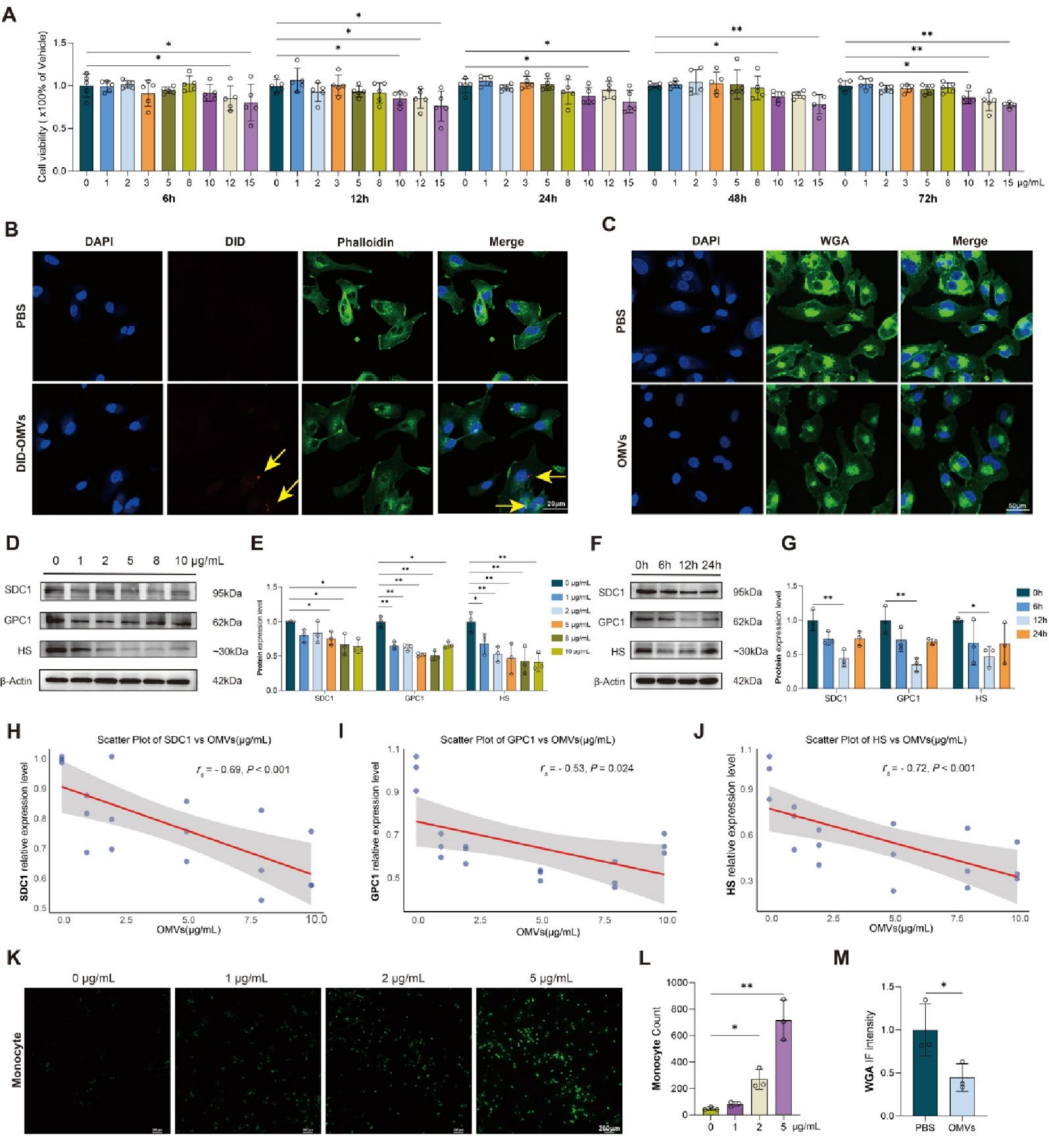
*P. gingivalis* OMVs promoted the glycocalyx injury of VECs and the adhesion of monocytes to VECs (**A**) Cell viability of EA. hy926 cells co-cultured with *P. gingivalis* OMVs for 6, 12, 24, 48 and 72 h; (**B**) IF staining showing the colocalization of DID-labeled OMVs in the cells; (**C**) IF staining showing changes in glycocalyx (WGA) of EA. hy926 cells after stimulation with OMVs for 12 h; (**D**) Immunoblots of SDC1, GPC1 and HS in EA. hy926 cells treated with different concentrations *P. gingivalis* OMVs (0, 1, 2, 5, 8, 10 µg/mL); (**E**) Quantitative analysis of SDC1, GPC1 and HS in EA. hy926 cells treated with *P. gingivalis* OMVs for different concentrations; (**F**) Immunoblots of SDC1, GPC1 and HS in EA. hy926 cells treated with *P. gingivalis* OMVs for 0, 6, 12, and 24 h; (**G**) Quantitative analysis of SDC1, GPC1 and HS in EA. hy926 cells treated with *P. gingivalis* OMVs for 0, 6, 12, 24 h; (**H**-**J**) Scatter plots of correlations between relative expression levels of SDC1, GPC1, HS and different concentrations of OMVs, respectively; (**K**) IF staining showing the changes of monocytes adhesion to EA. hy926 cells after stimulation with *P. gingivalis* OMVs for 12 h; (**L**) Monocyte (THP-1) count; (**M**) Relative fluorescent intensity (density) of WGA. (*n* ≥ 3, *P < 0.05, **P < 0.01). *Abbreviation* DAPI, 4’,6-diamidino-2-phenylindole; DID, 1,1’-dioctadecyl-3,3,3’,3’-tetramethylindodicarbocyanine,4-chlorobenzenesulfonate salt; GPC1, glypican-1; HS, heparan sulfate; IF, immunofluorescence; OMVs, outer membrane vesicles; SDC1, syndecan-1; WGA, wheat germ agglutinin

*P. gingivalis* W83 was cultured and *P. gingivalis* OMVs were successfully extracted and identified. TEM showed that the OMVs were circular, and NTA showed that the average diameter of the OMVs was approximately 129.8 nm (Figure S1A, B). At five time points including 6 h, 12 h, 24 h, 36 h, 48 h, and 72 h, the CCK8 assay revealed that *P. gingivalis* OMVs significantly inhibited proliferation of VECs (EA. hy926) only at concentration ≥ 10 µg/mL compared to that in the control group (Fig. 2A). We stimulated VECs with DID-labeled *P. gingivalis* OMVs at a concentration that does not affect the activity of VECs, and observed that the OMVs were taken up into the cell interior under confocal microscopy (Fig. 2B). We explored the optimal concentration of *P. gingivalis* OMVs and the time required to induce glycocalyx injury in endothelial cells and found that SDC1, GPC1, and HS, originating from the three main components of the glycocalyx, were significantly reduced when the concentration was ≥ 5 µg/mL (Fig. 2D, E). The expression levels of SDC1, GPC1, and HS demonstrated a significant inverse correlation with increasing concentrations of OMVs (Fig. 2H-J). The most obvious decrease in SDC1, GPC1, and HS was observed when VECs were exposed to *P. gingivalis* OMVs for 12 h (Fig. 2F, G). Using WGA to label the whole glycocalyx of VECs, we found that the fluorescence intensity of WGA was significantly decreased after stimulation with *P. gingivalis* OMVs for 12 h (Fig. 2C, M). Meanwhile, we found that the quantity of monocytes adhering to VECs was significantly enhanced after glycocalyx injury (Fig. 2K, L).

**Fig. 3:**
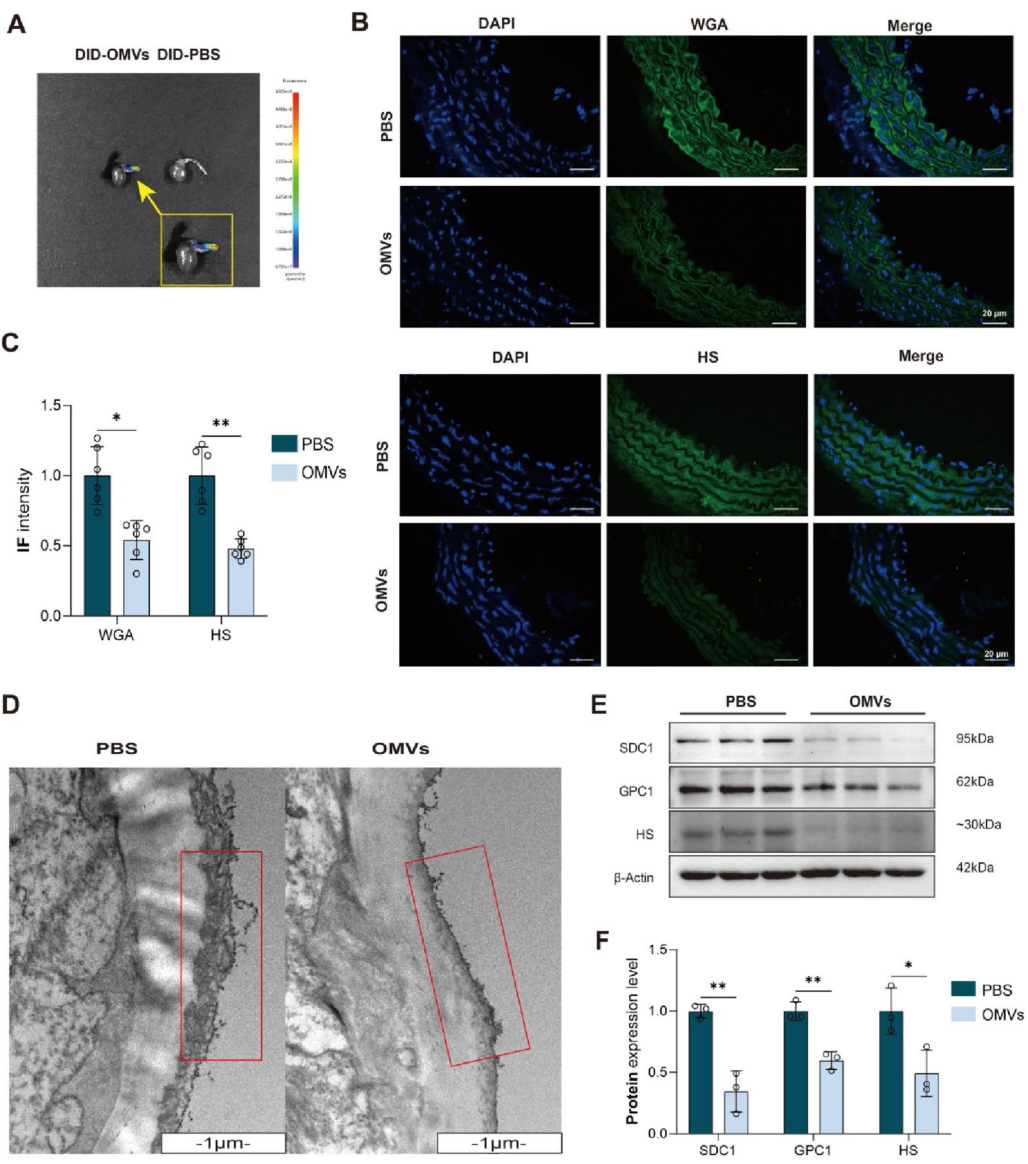
*P. gingivalis* OMVs promoted vascular endothelial glycocalyx injury in mice (**A**) Multimodal in vivo imaging of animals showing the concentration of DID-labeled OMVs in in the mouse arterial arch; (**B**) IF staining showing changes of vascular endothelial glycocalyx (WGA and HS) in mice after injection of *P. gingivalis* OMVs for 1 month through the tail vein; (**C**) Relative fluorescent intensity (density) of WGA and HS; (**D**) TEM showing morphology changes of glycocalyx in the aorta of mice after injected with *P. gingivalis* OMVs for 1 month through the tail vein, lanthanum nitrate staining revealed electron-dense glycocalyx clusters adherent to the endothelial luminal surface in the PBS group, the OMVs group demonstrated significant glycocalyx attenuation, with markedly diminished coverage on the endothelial surface; (**E**) Immunoblots of SDC1, GPC1 and HS in the aorta of mice after injection of *P. gingivalis* OMVs for 1 month through the tail vein; (**F**) Quantitative analysis of SDC1, GPC1 and HS in the aorta of mice after injection of *P. gingivalis* OMVs for 1 month through tail vein. (*n* ≥ 3, *P < 0.05, **P < 0.01). *Abbreviation* DAPI, 4’,6-diamidino-2-phenylindole; DID, 1,1’-dioctadecyl-3,3,3’,3’-tetramethylindodicarbocyanine,4-chlorobenzenesulfonate salt; GPC1, glypican-1; HS, heparan sulfate; IF, immunofluorescence; OMVs, outer membrane vesicles; SDC1, syndecan-1; WGA, wheat germ agglutinin

In the animal experiments, DID-labeled *P. gingivalis* OMVs were injected into the tail vein of C57B/6J mice. After 2 h, we observed high fluorescence intensity at the aortic arch under the in vivo imaging instrument (Fig. 3A). The mouse aorta was dissected, the proximal aortic arch tissue was cut for animal tissue section, and the remaining aorta tissue was used for WB experiment (Figure S2A-C). IF showed that the fluorescence intensity of glycocalyx labeled by HS and WGA in the *P. gingivalis* OMVs group was significantly decreased compared with that in PBS group (Fig. 3B, C). WB also confirmed that the protein of SDC1, GPC1, and HS was significantly reduced in the *P. gingivalis* OMVs group compared with the PBS group (Fig. 3E, F). TEM showed that the glycocalyx thickness in the *P. gingivalis* OMVs group was significantly thinner than that in the PBS group (Fig. 3D).

### *P. gingivalis* OMVs promoted vascular endothelial glycocalyx injury by inhibiting the expression of B3GAT1

**Fig. 4:**
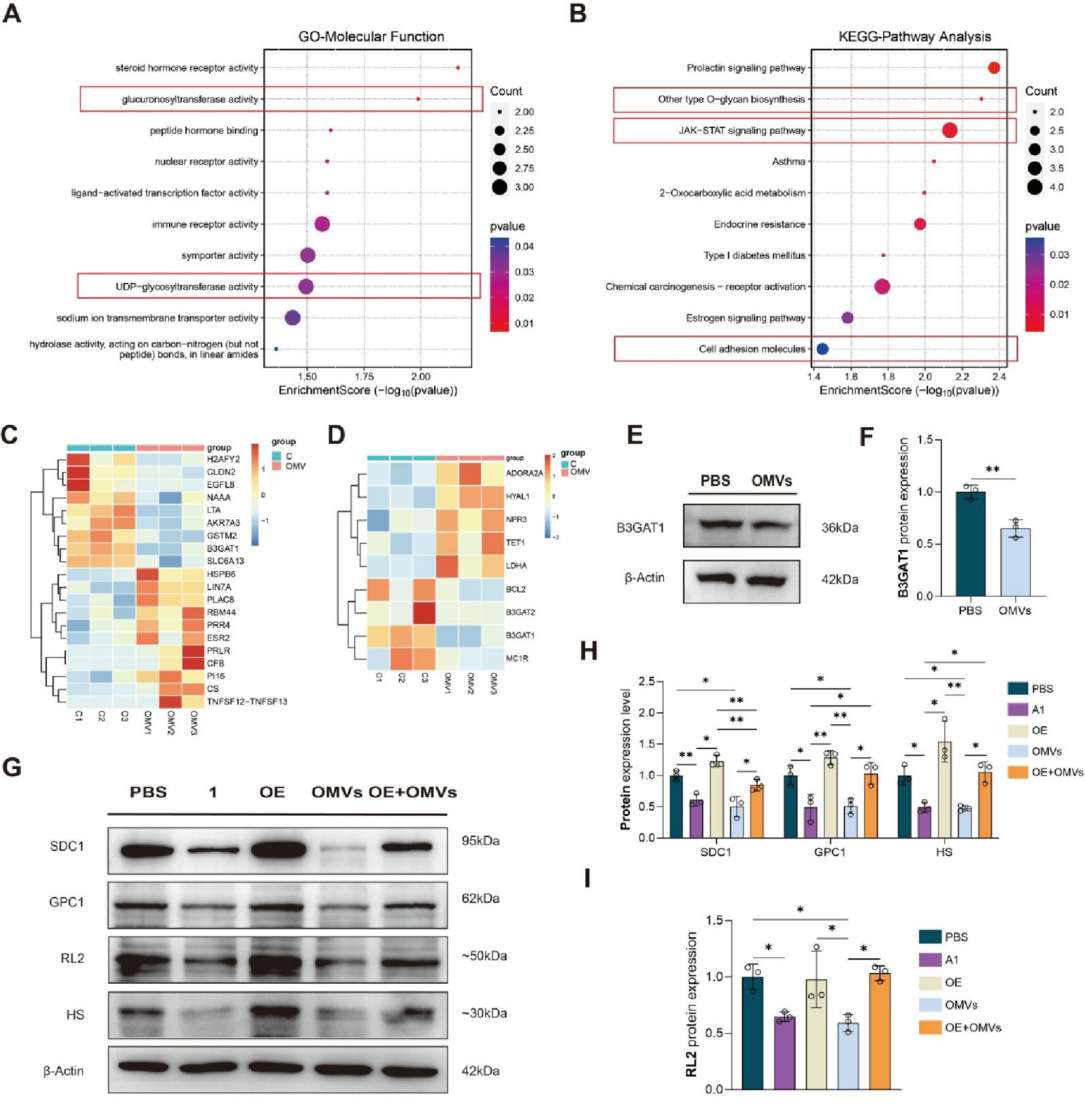
*P. gingivalis* OMVs promoted vascular endothelial glycocalyx injury by inhibiting the expression of B3GAT1 (**A**) Scatter diagram of significantly enriched functions, such as glucuronosyltransferase activity; (**B**) Scatter diagram of significantly enriched pathways, such as other type O-glycan biosynthesis, JAK-STAT signaling pathway and cell adhesion molecules; (**C**) Heatmap of differential genes involved in the carbohydrate derivative biosynthetic process; (**D**) Heatmap of differential genes involved in the extracellular region; (**E**) Immunoblots of B3GAT1 in EA. hy926 cells treated with *P. gingivalis* OMVs; (**F**) Quantitative analysis of B3GAT1 in EA. hy926 cells treated with *P. gingivalis* OMVs; (**G**) Immunoblots of SDC1, GPC1, HS and RL2 of EA. hy926 cells in cellular rescue experiments; (**H**, **I**) Quantitative analysis of SDC1, GPC1, HS and RL2 of EA. hy926 cells in rescue assays. (*n* = 3, *P < 0.05, **P < 0.01). *Abbreviation* B3GAT1, β 1,3-glucuronosyltransferase; GPC1, glypican-1; HS, heparan sulfate; IF, immunofluorescence; OE, over expression; OMVs, outer membrane vesicles; RL2, O-Linked N-Acetylglucosamine; SDC1, syndecan-1; WGA, wheat germ agglutinin

Through high-throughput sequencing of mRNA levels of EA. hy926 cells stimulated by *P. gingivalis* OMVs and PBS, a total of 13, 460 core genes were expressed between samples, as well as 126 differentially expressed genes, including 72 upregulated genes and 54 downregulated genes (Figure S3A, B). The volcano map of differential expression between groups and the cluster heat map of differential gene expression are shown in Figure S3C, D. GO function enrichment analysis and KEGG pathway enrichment analysis were performed on the differential genes. The top 20 GO function enrichment pathways included glucuronosyltransferase activity, chondroitin sulfate proteoglycan metabolic process, and chondroitin sulfate metabolic process (top 10 of these were shown in Fig. 4A). The top 20 KEGG pathway enrichment included other types of O−glycan biosynthesis and glycosaminoglycan degradation (top10 were shown in Fig. 4B, all pathways were shown in Figure S4A). We selected the differentially expressed gene *B3GAT1* (log2|FC| = 8.90, *P* = 0.025), which is closely related to the synthesis and degradation of glycosaminoglycan, to investigate its role in vascular endothelial glycocalyx injury caused by *P. gingivalis* OMVs. We found that B3GAT1 is both involved in the carbohydrate derivative biosynthetic process and the extracellular region, as demonstrated in the relevant heat maps shown in Fig. 4C, D.

**Fig. 5:**
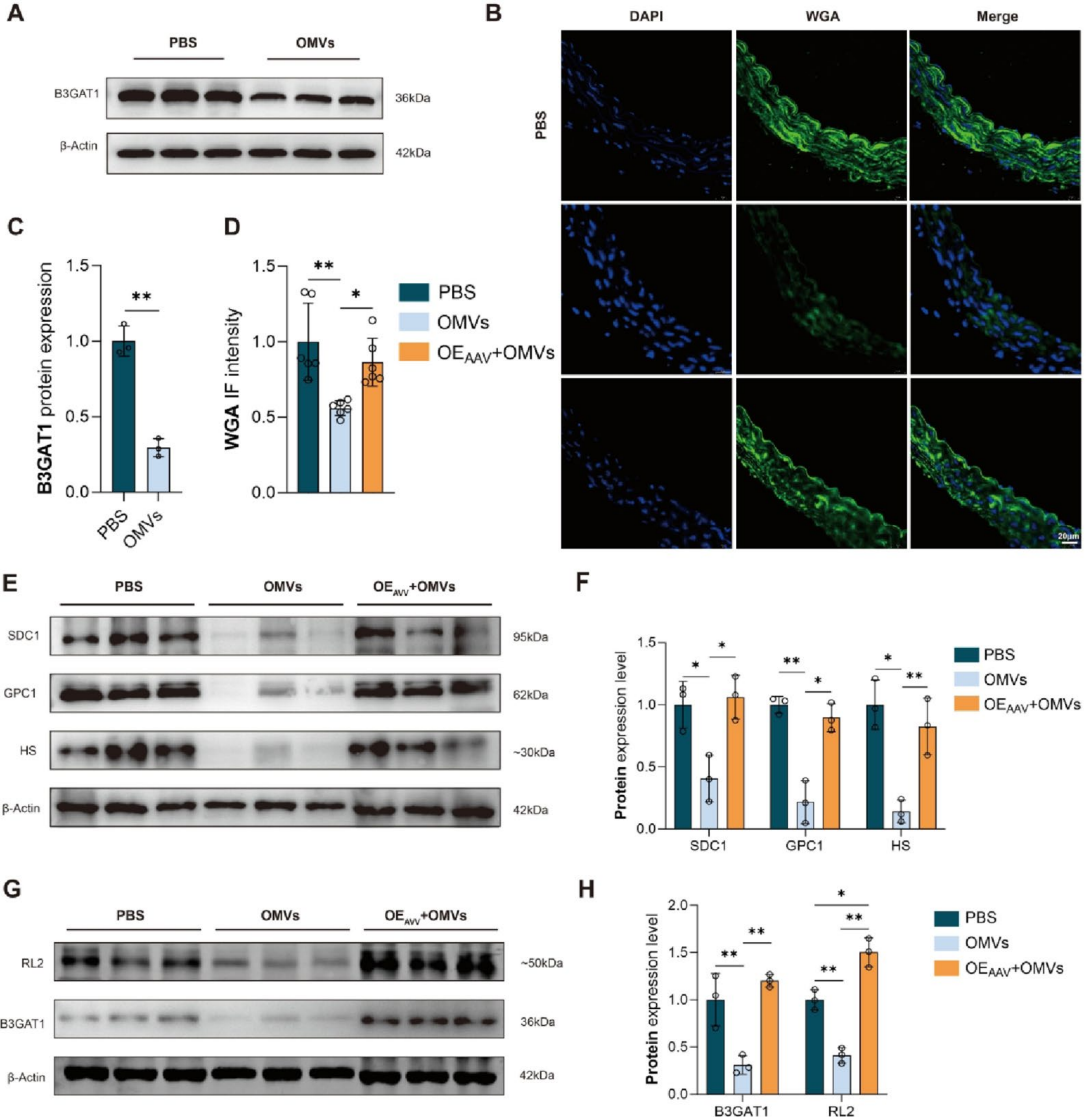
In vivo rescue experiments (**A**) Immunoblots of B3GAT1 in the aorta of mice after injection of *P. gingivalis* OMVs for 1 month through the tail vein; (**B**) IF staining showing the discrepancy of glycocalyx (WGA) in the PBS, OMVs, and OE_AAV_+OMVs group; (**C**) Quantitative analysis of B3GAT1 in the aorta of mice after injection of *P. gingivalis* OMVs for 1 month through the tail vein; (**D**) Relative fluorescent intensity (density) of WGA in the aorta of mice in PBS, OMVs, and OE_AAV_+OMVs group; (**E**) Immunoblots of SDC1, GPC1 and HS in the aorta of mice in the PBS, OMVs, and OE_AAV_+OMVs group; (**F**) Quantitative analysis of SDC1, GPC1 and HS in the aorta of mice in the PBS, OMVs, and OE_AAV_+OMVs group; (**G**) Immunoblots of B3GAT1 and RL2 in the aorta of mice in the PBS, OMVs, and OE_AAV_+OMVs group; (**H**) Quantitative analysis of B3GAT1 and RL2 in the aorta of mice in the PBS, OMVs, and OE_AAV_+OMVs group. (*n* ≥ 3, *P < 0.05, **P < 0.01). *Abbreviation* AAV, adeno-associate virus; B3GAT1, β 1,3-glucuronosyltransferase; DAPI, 4’,6-diamidino-2-phenylindole; GPC1, glypican-1; HS, heparan sulfate; IF, immunofluorescence; OE, over expression; OMVs, outer membrane vesicles; RL2, O-Linked N-Acetylglucosamine; SDC1, syndecan-1; WGA, wheat germ agglutinin

Next, we verified the sequencing results. RT-qPCR showed that the expression level of *B3GAT1* mRNA in the OMVs group was significantly lower than that in the PBS group (Figure S5A), while there were no differences on the mRNA level of *BCL2*, *ESR2*, *IRF9* and *PRLR* in the two groups (Figure S5B-E). WB results also showed that the expression of B3GAT1 protein in the OMVs group was significantly lower than that in the PBS group in EA. hy926 cells (Fig. 4E, F), which was also observed with the in vivo experiments (Fig. 5A, C).

To verify the role of B3GAT1 in glycocalyx injury, we constructed siRNA that can interfere with the expression of B3GAT1 in cells, named A1, A2, and A3, respectively, and constructed plasmid that can make cells overexpress B3GAT1, named OE (verified as Figure S6A). The transfection efficiency of siRNA was verified, and it was observed that siRNA A1 could significantly reduce the expression of B3GAT1 protein in EA. hy926 cells to less than 25% (Figure S7A-C). By verifying the transfection efficiency of OE, we observed that the plasmid OE could significantly increase the expression of B3GAT1 protein in EA. hy926 cells over 2.2 times (Figure S8A-C). After transfecting the cells with siRNA-A1 and OE, the rescue experiment showed that the expressions of SDC1, GPC1, and HS in the A1 and OMVs groups were significantly reduced compared with those in the PBS group, whereas the expressions of SDC1, GPC1, and HS in the OE + OMVs group were significantly increased compared with those in the A1 and OMVs group (*P* < 0.05) (Fig. 4G, H). AS B3GAT1 was an important glycosyltransferase, we examined the glycosylation indicators and found that the expression trend of RL2 was the same as that of SDC1, GPC1, and HS (Fig. 4G, I).

To determine the role of B3GAT1 in glycocalyx injury in vivo, we constructed AAV overexpressing B3GAT1 and transfected C57B/6J mice through tail vein injection to create transgenic mice. One week after injection, one mouse from each of the PBS, NC_AAV_, and OE_AAV_ groups was euthanized to verify the successful construction of our transgenic mice and confirm B3GAT1 overexpression in the aorta. IF showed that Flag protein was expressed in the aorta of both the NC_AAV_ and OE_AAV_ groups (Figure S9A). Meanwhile, WB test showed that the OE_AAV_ group exhibited positive expression of Flag, and the expression of B3GAT1 protein was significantly increased (Figure S9B, C). Aorta tissue was collected one month after tail vein injection of *P. gingivalis* OMVs. IF revealed that the fluorescence intensity of WGA in the OMVs group was notably weaker than that in the PBS group. Conversely, the fluorescence intensity of WGA in the OE_AAV_+OMVs group was significantly higher than that in the OMVs group. However, there was no significant difference between the PBS group and the OE_AAV_+OMVs group (Fig. 5B, D). WB experiments also showed that the expression levels of SDC1, GPC1, and HS in the OMVs group were significantly decreased compared with the PBS group, while the expression levels of SDC1, GPC1, and HS in the OE_AAV_+OMVs group were significantly increased compared with OMVs group. There was no significant difference in SDC1, GPC1, and HS expression between the PBS and OE_AAV_+OMVs group (Fig. 5E, F). WB further revealed that the expression levels of B3GAT1 and RL2 in the PBS and OE_AAV_+OMVs group were higher than those in the OMVs group (Fig. 5G, H).

### PPAD in *P. gingivalis* OMVs promoted vascular endothelial glycocalyx injury by inhibiting B3GAT1 expression

To examine the specific virulence factors in *P. gingivalis* OMVs, we successfully cultured and extracted the OMVs of ΔPG1424 (obtained our previous experiment). The results of TEM were shown in Figure S10A**.** NTA showed that the average diameters of the *P. gingivalis* ^ΔPPAD^OMVs was 133.9 nm (Figure S10B).

**Fig. 6:**
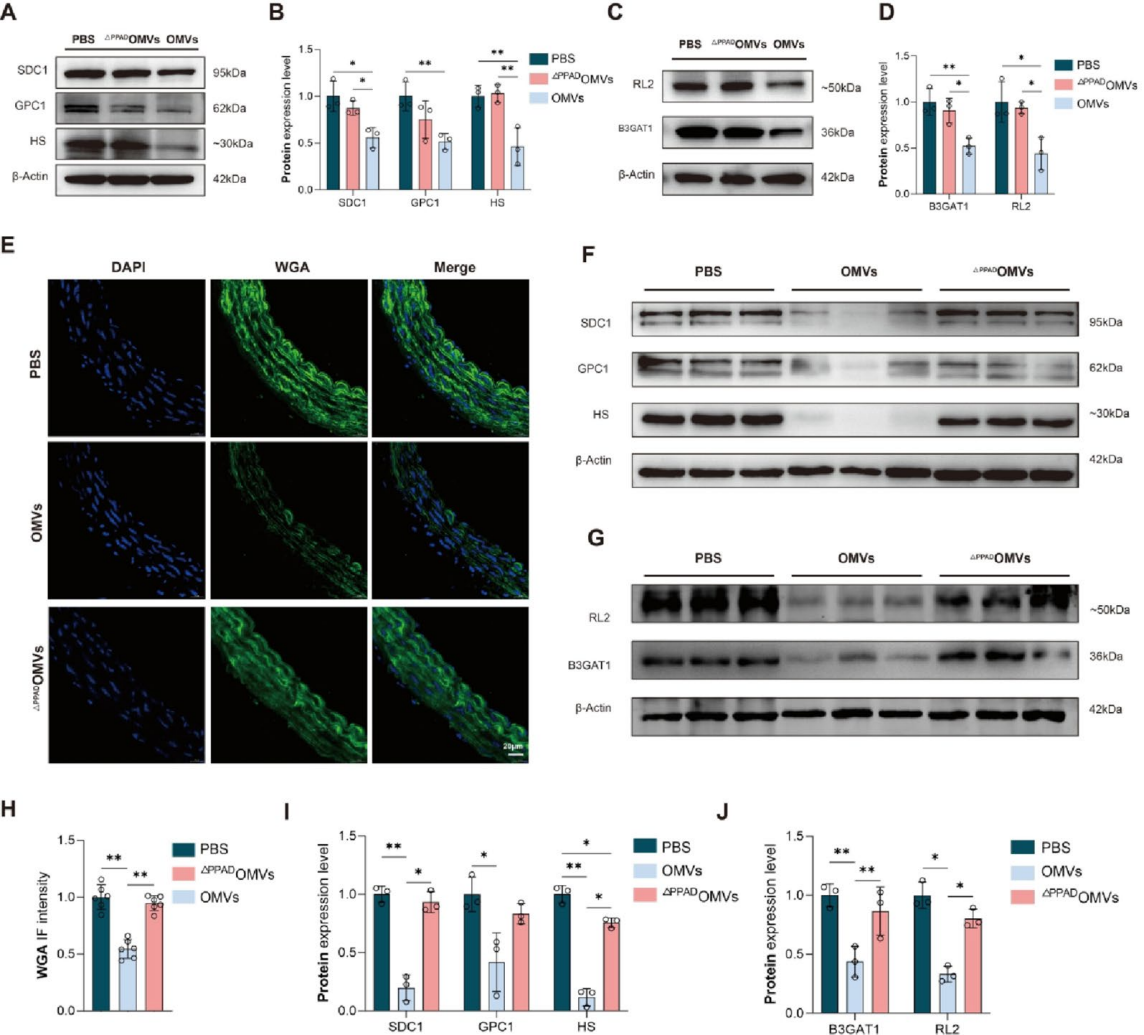
PPAD in *P. gingivalis* OMVs promoted vascular endothelial glycocalyx injury by inhibiting B3GAT1 expression (**A**) Immunoblots of SDC1, GPC1 and HS in EA. hy926 cells treated with PBS, *P. gingivalis* ^ΔPPAD^OMVs and *P. gingivalis* OMVs; (**B**) Quantitative analysis of SDC1, GPC1 and HS in EA. hy926 cells treated with PBS, *P. gingivalis* ^ΔPPAD^OMVs and *P. gingivalis* OMVs; (**C**) Immunoblots of B3GAT1 and RL2 in EA. hy926 cells treated with PBS, *P. gingivalis* ^ΔPPAD^OMVs and *P. gingivalis* OMVs; (**D**) Quantitative analysis of B3GAT1 and RL2 in EA. hy926 cells treated with PBS, *P. gingivalis* ^ΔPPAD^OMVs and *P. gingivalis* OMVs; (**E**) IF staining showing changes of vascular endothelial glycocalyx in mice after injection of PBS, *P. gingivalis* OMVs and *P. gingivalis* ^ΔPPAD^OMVs for 1 month through the tail vein; (**F**) Immunoblots of SDC1, GPC1 and HS in the aorta of mice in the PBS, OMVs and, ^ΔPPAD^OMVs group; (**G**) Immunoblots of B3GAT1 and RL2 in the aorta of mice in the PBS, OMVs and, ^ΔPPAD^OMVs group; (**H**) Relative fluorescent intensity (density) of WGA in the aorta of mice in the PBS, OMVs and, ^ΔPPAD^OMVs group (corresponding to **E**); (**I**) Quantitative analysis of SDC1, GPC1 and HS in the aorta of mice in the PBS group, OMVs and, ^ΔPPAD^OMVs group; (**J**) Quantitative analysis of B3GAT1 and RL2 in the aorta of mice in the PBS, OMVs, and ^ΔPPAD^OMVs group. (*n* ≥ 3, *P < 0.05, **P < 0.01). *Abbreviation* B3GAT1, β 1,3-glucuronosyltransferase; DAPI, 4’,6-diamidino-2-phenylindole; GPC1, glypican-1; HS, heparan sulfate; IF, immunofluorescence; OE, over expression; OMVs, outer membrane vesicles; RL2, O-Linked N-Acetylglucosamine; SDC1, syndecan-1; WGA, wheat germ agglutinin

Among in vitro experiments, WB showed that the expression levels of SDC1, GPC1, and HS in the OMVs group were significantly lower than those in the PBS group, whereas only the expression levels of SDC1 and HS in the OMVs group were significantly lower than those in the ^ΔPPAD^OMVs group. There were no significant differences in SDC1, GPC1, and HS expression levels between the PBS and ^ΔPPAD^OMVs groups (Fig. 6A, B). Similarly, WB showed that the expression levels of B3GAT1 and RL2 in the OMVs group were significantly decreased compared with those in the PBS group and ^ΔPPAD^OMVs groups, while there was no significant difference in the expression levels of B3GAT1 and RL2 between the PBS and ^ΔPPAD^OMVs group (Fig. 6C, D).

Additionally, with the in vivo experiments, the fluorescence intensity of WGA in the OMVs group was significantly decreased than that in the PBS and ^ΔPPAD^OMVs groups. However, there was no significant difference in the fluorescence intensity of WGA between the PBS and ^ΔPPAD^OMVs group (Fig. 6E, H). WB showed that the expression levels of SDC1, GPC1, and HS in the OMVs group were significantly lower than those in the PBS group, while the expression levels of SDC1 and HS in the OMVs group were significantly lower than those in the ^ΔPPAD^OMVs group. There were no significant differences in expression levels between the PBS and ^ΔPPAD^OMVs groups (Fig. 6F, I). Similarly, WB showed that the expression levels of B3GAT1 and RL2 in the OMVs group were significantly decreased compared with those in the PBS and ^ΔPPAD^OMVs groups, while there was no significant difference in the expression levels of B3GAT1 and RL2 between the PBS and ^ΔPPAD^OMVs groups (Fig. 6G, J).

### Function exploration of PPAD in ***P. gingivalis*** OMVs

**Fig. 7:**
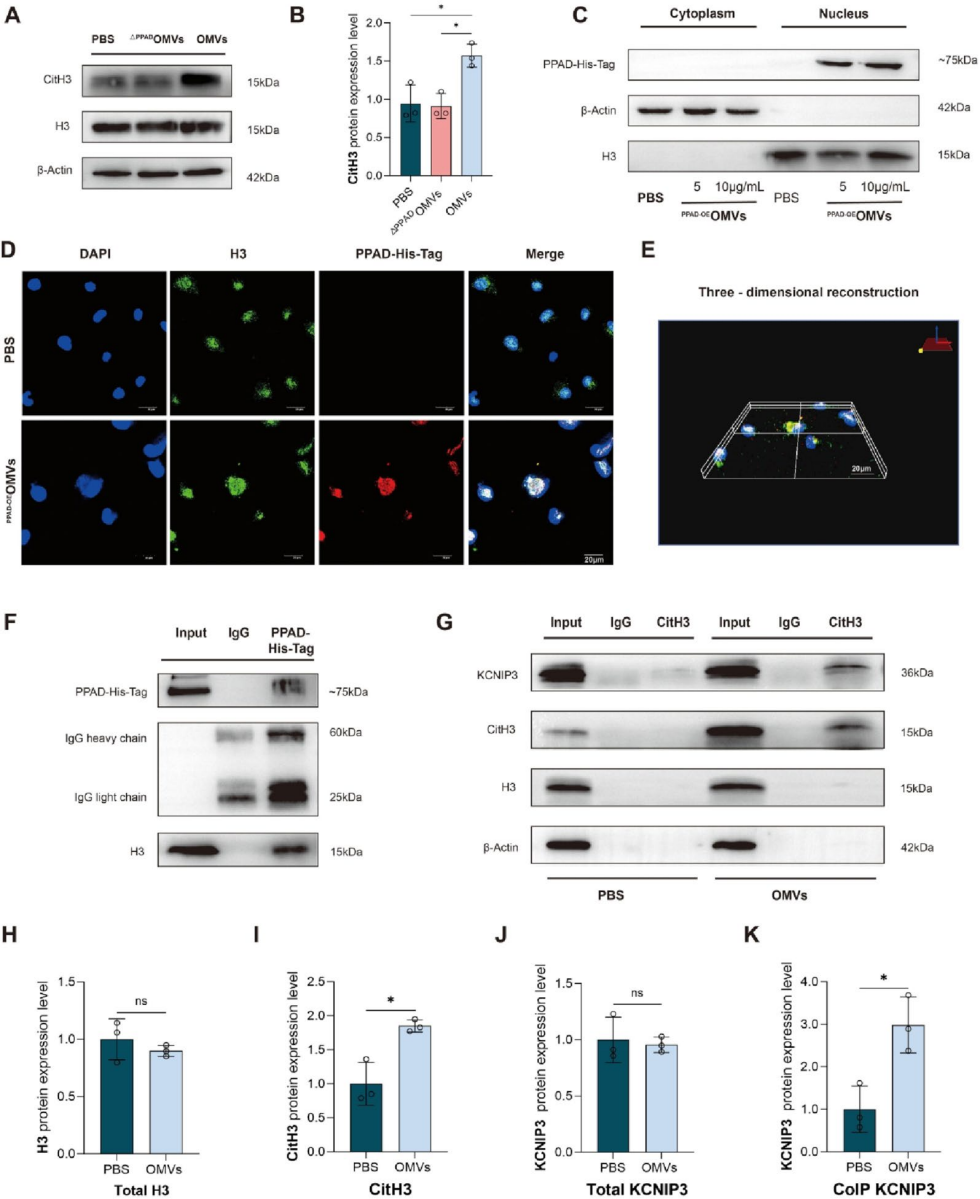
PPAD could enter the nucleus of EA. hy926 cells and induce citrullination of histone H3 (**A**) Immunoblots of CitH3 in EA. hy926 cells treated with PBS, *P. gingivalis* ^ΔPPAD^OMVs and *P. gingivalis* OMVs; (**B**) Quantitative analysis of CitH3 in EA. hy926 cells treated with PBS, *P. gingivalis* ^ΔPPAD^OMVs and *P. gingivalis* OMVs; (**C**) Immunoblots of PPAD-His-Tag in Nucleus of EA. hy926 cells treated with *P. gingivalis* ^PPAD−OE^OMVs; (**D**,** E**) IF staining showing the colocalization of PPAD-His-Tag with histone H3; (**F**) Immunoblots of PPAD-His-Tag and histone H3 in EA. hy926 cells treated with *P. gingivalis* ^PPAD−OE^OMVs, CoIP showing the interaction between PPAD and histone H3, and immunoprecipitation with His-Tag antibody; (**G**) Immunoblots of KCNIP3, histone H3, and CitH3 in EA. hy926 cells treated with PBS and *P. gingivalis* OMVs, expression levels of total KCNIP3 and histone H3 showing no significant differences between OMVs and PBS groups, immunoprecipitation with CitH3 antibody detecting markedly elevated KCNIP3 levels in the OMVs group; (**H**-**K**) Quantitative analysis of KCNIP3, histone H3, and CitH3 in EA. hy926 cells treated with PBS and *P. gingivalis* OMVs. (*n* = 3, *P < 0.05). *Abbreviation* B3GAT1, β 1,3-glucuronosyltransferase; CitH3, citrullinated histone H3; CoIP, Co-Immunoprecipitation; DAPI, 4’,6-diamidino-2-phenylindole; GPC1, glypican-1; HS, heparan sulfate; IF, immunofluorescence; KCNIP3, Kv channel interacting protein 3; OE, over expression; OMVs, outer membrane vesicles; PPAD, *Porphyromonas gingivalis* peptidylarginine deiminase; RL2, O-Linked N-Acetylglucosamine; SDC1, syndecan-1; WGA, wheat germ agglutinin

As a key protein of nucleosomes in the nucleus, histone H3 can undergo various covalent modifications, such as acetylation, methylation, phosphorylation, ubiquitination, and citrullination. These modifications significantly affect the regulation of gene expression [24]. Therefore, we aimed to verify whether PPAD in *P. gingivalis* OMVs induces citrullination of histone H3, leading to a reduction in B3GAT1 expression and subsequently promoting vascular endothelial glycocalyx injury. In the following experiment, we found that the expression of citrullinated histone H3 (CitH3) in the OMVs group was significantly higher than that in the PBS and ^ΔPPAD^OMVs groups (Fig. 7A, B). We transfected the overexpressed PPAD plasmid carrying a His-Tag into *P. gingivalis* W83 using bacterial electroporation, obtaining a strain expressing PPAD-His-Tag. Then, we cultured and extracted ^PPAD−OE^OMVs. Agarose gel electrophoresis was used to verify the successful transfection of the strain (Figure S11A).

A prerequisite for the citrullination of H3 by PPAD is the entrance of PPAD into the nucleus. We successfully extracted the cytoplasmic protein and nucleoprotein of EA. hy926 cells stimulated using PBS and ^PPAD−OE^OMVs respectively. WB results showed that PPAD-His-Tag could be detected in nucleoprotein (Fig. 7C). Through IF experiments, we observed that PPAD can enter the cell nucleus and co-localize with histone H3 (Fig. 7D, E). Molecular dynamics simulations revealed critical hydrogen bonding and salt bridge interactions between PPAD and histone H3 at key catalytic residues (Figure S12, Table S3). Co-immunoprecipitation (CoIP) experiment further verified the interaction between PPAD and H3 (Fig. 7F). LC-MS/MS analysis of immunoprecipitated complexes using His-Tag antibodies identified 222 proteins in the His-Tag group versus 250 proteins in the IgG control. Histone H3 was detected in both groups, with intensity-based absolute quantification (iBAQ) values revealing a 23.8-fold enrichment in the His-Tag group (iBAQ = 9,918,900) compared to the IgG group (iBAQ = 416,220), demonstrating a specific interaction between PPAD and histone H3 (Figure S13).

Through ChEA3 database analysis predicting transcriptional regulators of B3GAT1, KCNIP3 was identified as the top-ranked transcriptional repressor (Figure S14). Molecular docking simulations demonstrated stable binding interactions between histone H3/KCNIP3 and the transcriptional regulatory region of B3GAT1, with confidence scores exceeding 90%, indicating robust binding stability (Figure S15; Table S4). EA. hy926 cells were stimulated with OMVs subsequent experiments and CoIP assays revealed that total KCNIP3 and histone H3 expression levels showed no significant differences between OMVs and PBS groups. However, immunoprecipitation with the CitH3 antibody detected markedly elevated KCNIP3 levels in OMV-treated samples (Fig. 7G-K). These findings suggested that PPAD in OMVs induced histone H3 citrullination modification, enabling CitH3 to recruit the transcriptional repressor KCNIP3. This interaction ultimately suppresses B3GAT1 expression, resulting in vascular endothelial glycocalyx injury.

## Discussion

In 1966, Luft [25] first described the glycocalyx as a fluffy structure of approximately 20 nm in size, and present on the surface of endothelial cells, observed using electron microscopy technology. With the development of visualization techniques and in-depth research, understanding about the structure and composition of the glycocalyx has increased gradually. The glycocalyx exhibits a barrier function and selective permeability, regulating fluid flow across blood vessels, sensing fluid shear force, transmitting guidance signals to the cytoskeleton of endothelial cells, and modulating NO-mediated vasodilation to control blood pressure. Additionally, the glycocalyx endows the surface of endothelial cells with anticoagulant and antiadhesive properties, thereby preventing the formation of thrombus on the vascular endothelial cell surface [9, 12, 26]. Glycocalyx homeostasis is closely linked to the functional stability of blood vessels. Disruption of the glycocalyx structure has been observed in various diseases, including diabetes, AS, sepsis, ischemia/reperfusion injury, chronic/acute kidney diseases and viral infections, such as COVID-19 [27–32]. Conversely, the intact vascular endothelial glycocalyx also serves as a natural barrier against pathogens such as viruses [33]. Abassi et al. [14] showed that the circulating levels of SDC1 and HS, as well as other glycocalyx components, can be used as effective biomarkers to monitor the severity of vascular endothelial injury, and can serve as targets for the treatment of AS. Furthermore, studies have indicated that HA promotes the activation, proliferation, and migration of vascular smooth muscle cells, making it a useful auxiliary indicator for assessing the severity of AS [34]. Therefore, glycocalyx shedding is of great significance for the monitoring, diagnosis, and treatment of cardiovascular diseases such as AS. In our experiment, we were surprised to discover that the serum levels of SDC1 and HS in patients with stage III-IV periodontitis were significantly elevated compared to those in participants with no/stage I-II periodontitis. This is also the first study to explore the correlation between glycocalyx and periodontitis. In the subsequent analysis, we found a strong correlation between the periodontal status-related indices PPD, CAL, and BOP and serum levels of SDC1 and HS. The levels of SDC1 and HS increased with an increase in these periodontal indices, indicating that the damage to the vascular endothelial glycocalyx progressively worsened with the severity of periodontitis. Subsequently, we employed ROC curve analysis to demonstrate the potential utility of serum SDC1 and HS as biomarkers for evaluating severity and progression of periodontitis. Our experiment provided new evidence about the impact of periodontitis on CVDs, aiming to suggest novel strategies for enhancing the prevention and treatment of CVDs.

As the primary periodontal pathogen, *P. gingivalis* and the toxic virulence factors it releases can trigger a series of immune responses in the body, resulting in the entry of mediators into circulation blood. These series of events can subsequently influence the onset and progression of CVDs [4, 35]. *P. gingivalis* can also release small, adherent, and stable OMVs, which can be internalized into host tissues and activate proinflammatory pathways in host cells [16]. Due to the protection provided by the vesicle membrane structure, high concentrations of pathogenic factors can evade degradation and facilitate long-distance transmission, thereby increasing the virulence of *P. gingivalis* OMVs compared to that of other bacteria [36]. Farrugia et al. [16, 17] demonstrated that *P. gingivalis* OMVs could hydrolyze and cleave human VECs through gingival protease, leading to increased vascular permeability, which in turn results in increased tissue exudate and tissue edema. Other studies have shown that *P. gingivalis* OMVs, by inhibiting the function of NO, causes increased vasoconstriction and elevated endothelial cell apoptosis, and promotes the development of AS [18, 19]. *P. gingivalis* OMVs could also aggravate diabetic retinopathy, increase the expression of inflammatory factors in retinal microvascular endothelial cells, production of reactive oxygen species, and cause mitochondrial dysfunction, thus inducing endothelial cell dysfunction [22]. In our preliminary experiments, we systematically compared the differential effects of three principal virulence components of *P. gingivalis*, including lipopolysaccharide (LPS), gingipains, and OMVs on endothelial glycocalyx integrity. Notably, our quantitative analyses revealed that OMVs demonstrated significantly greater disruptive potential compared to LPS and gingipains. This conclusion was substantiated through two complementary methodologies.WB analysis confirmed the significant downregulation of two critical glycocalyx structural components of SDC1 and HS, specifically in OMVs-exposed endothelial cells (Figure S16). Subsequent IF staining with FITC-WGA labeling further showed a marked reduction in overall glycocalyx fluorescence intensity in the OMVs-treated groups (Figure S17). To further validate our findings, we compared the effects of *P. gingivalis* OMVs with those of *P. endodontalis* OMVs, another major oral pathogen, on vascular endothelial glycocalyx integrity. WB analysis revealed that *P. gingivalis* OMVs caused significantly greater glycocalyx injury (Figure S18). Based on these compelling preliminary findings, we innovatively selected *P. gingivalis*-derived OMVs as the primary pathogenic mediator for subsequent mechanistic investigations.

In two studies by Andrian et al., gingival epithelial cells were stimulated with *P. gingivalis* LPS and supernatant from a gingipain - deficient mutant, resulting in increased SDC1 concentration in the culture supernatant [37, 38]. In two studies by Smith et al., changes in chondroitin sulfate in gingival crevicular fluid of periodontitis patients were observed, and in vitro stimulation of gingival epithelial cells with *P. gingivalis* supernatant resulted in increased HS concentration in the supernatant [39, 40]. These periodontal - related studies indicated that LPS and gingipains could elevate SDC1 and HS concentrations in culture supernatants via proinflammatory pathways; however, none have directly concluded that LPS and gingipains degrade SDC1 and HS, which is consistent with previous research suggesting that glycocalyx degradation occurs via specific enzymes (heparanase, hyaluronidase and matrix metalloproteinase) [41]. The aforementioned studies have mainly focused on the effects of *P. gingivalis* on glycocalyx components in local periodontal settings. As the relationship between periodontitis, periodontal pathogens and systemic diseases is gaining attention, our study innovatively focused on the impact of periodontitis and *P. gingivalis* on systemic diseases. We investigated the effects of periodontitis and *P. gingivalis* on vascular endothelial glycocalyx, both overall and at the molecular level, making our study more comprehensive and systematic. In this study, we found that *P. gingivalis* OMVs can induce glycocalyx injury of VECs, both in vitro and in vivo. This is because the glycocalyx is a dynamically changing structure on the surface of the vascular endothelium, involving both degradation and synthesis processes. In vitro experiments showed that *P. gingivalis* OMVs caused the most significant damage to the glycocalyx at the 12 h time point (Fig. 2F, G), which was consistent with a previous study [42]. Furthermore, no glycocalyx injury was observed for short durations less than 6 h, indicating that the glycocalyx injury did not result from the direct degradation of *P. gingivalis* OMVs by gingival proteases. As shown in Fig. 2H-J, a dose-dependent reduction in key glycocalyx components (SDC1, GPC1, and HS) was observed with escalating concentrations of *P. gingivalis* OMVs, demonstrating that elevated inflammatory stimulation exacerbates glycocalyx injury. In vivo experiments revealed glycocalyx injury in C57BL/6J mice following tail vein administration of *P. gingivalis* OMVs. TEM demonstrated significant thinning and structural disruption of the aortic glycocalyx layer, providing morphological evidence of OMVs-induced glycocalyx injury. Furthermore, molecular analyses through aortic tissue IF and WB confirmed the downregulation of core glycocalyx components (SDC1, GPC1, and HS), mechanistically linking *P. gingivalis* OMVs exposure to glycocalyx injury.

Protein glycosylation, a post-translational modification, involves the enzymatic attachment of carbohydrate chains to specific residues on protein sequences. Glycosylation influences various aspects of protein function, such as protein folding, enzyme activity, and interactions between cells and the extracellular matrix [43]. B3GAT1 (β 1,3-glucuronosyltransferase) is a key enzyme in the regulation of glycosylation, also known as galactose-β 1.3-glucuronosyltransferase P (GIcAT-P), which belongs to the glycosyltransferase family [44]. Through the other-type glycosylation pathway, B3GAT1 is capable of producing glycosaminoglycans, which in turn regulate growth factors, cytokines, morphogens, and various protein ligands. These glycosaminoglycans plays a crucial role in cell proliferation, cell adhesion, coagulation, inflammation, and wound healing [45]. Further study showed that O-glycosylation was closely linked to the integrity of the glycocalyx, ensuring a robust barrier function [46]. Trimarco et al. [47] showed that glycosylation modification by B3GAT1 could broadly restrict influenza virus infection. Normal expression of B3GAT1 was beneficial to human health, while low expression of B3GAT1 can aggravate the development of related diseases [47, 48]. Our study also confirmed that there was a state of low B3GAT1 expression during the glycocalyx injury induced by *P. gingivalis* OMVs. Furthermore, overexpression of B3GAT1 in our cellular rescue experiments reduced the glycocalyx injury. Therefore, it was evident that the normal expression of B3GAT1 is crucial for maintaining the homeostasis of the glycocalyx. In in vivo rescue experiments, aortic-specific overexpression of B3GAT1 was achieved in C57BL/6J mice via tail vein injection of AAV-Flag-B3GAT1. This transgenic intervention significantly mitigated *P. gingivalis* OMVs-induced vascular endothelial glycocalyx injury, demonstrating the therapeutic potential of B3GAT1 restoration in preserving glycocalyx integrity. Additionally, our experiments showed that RL2, which reflects the level of glycosylation, exhibits a similar trend in its expression as B3GAT1. These findings collectively demonstrated that ​B3GAT1-mediated glycosylation and the associated ​increase in O-glycosylation levels played a critical role in preserving vascular endothelial glycocalyx integrity. Consequently, ​B3GAT1 might emerge as a promising therapeutic target for mitigating glycocalyx damage.

Peptidyarginine deaminase (PAD), a protein modifying enzyme, can modify and transform host proteins by converting peptidyl-arginine into peptidyl-citrulline, a process known as citrullination [49]. Protein citrullination can trigger abnormal autoimmune responses by altering the immune activity of chemokines, as well as modifying the structure and function of proteins [50, 51]. Studies have shown that citrullinated protein could be detected in the synovial tissue of patients with rheumatoid arthritis (RA), and PAD can also be found to be upregulated [52]. Other diseases related to citrullination include cancer, central nervous system diseases, inflammatory diseases, and immune disorders [53–55]. The upregulation of PAD has also been observed in a variety of CVDs, including myocardial infarction, AS, cardiac fibrosis, heart failure, and venous thrombosis [56–58]. A growing body of evidence has shown that PAD-mediated citrullination of histones regulates gene transcription and other processes [59, 60]. As the sole Gram-negative bacterium capable of producing PAD, PPAD produced by *P. gingivalis* shares similar structure and function with the PAD family members in the host. Additionally, PPAD lacked a regulatory cofactor that prevented citrullination, potentially resulting in unregulated and excessive citrullination modification of host proteins [61, 62]. In most cases, PPAD was abundantly present in secreted OMVs that are massively produced by *P. gingivalis*, and to a lesser extent in a soluble secreted state [63]. Currently, studies have demonstrated that PPAD is directly or indirectly involved in the processes of periodontitis, RA, AD, CVDs, AS, and other diseases [61, 64, 65]. In this study, we conducted experiments using OMVs and ^ΔPPAD^OMVs. Interestingly, only OMVs were capable of reducing glycocalyx component proteins, while ^ΔPPAD^OMVs were unable to cause glycocalyx injury due to the lack of PPAD. Notably, downregulation of B3GAT1 and RL2 expression was exclusively observed in both in vivo and in vitro OMV-treated groups, accompanied by reduced CitH3 levels. These findings constituted the first evidence that PPAD within OMVs modulated downstream gene expression in CVDs through citrullination of histone H3, addressing a critical knowledge gap in the field. Current studies use *​​Escherichia coli* LPS to stimulate and detect glycocalyx changes, demonstrating that it increases glycocalyx-degrading enzymes via TLRs, NF-κB and proinflammatory mechanisms [66, 67]. In contrast, our work employed *P. gingivalis*-derived OMVs containing PPAD and LPS as stimuli, revealing distinct endothelial glycocalyx injury. This differential effect may arise from the unique LPS composition within OMVs and the enriched PPAD content characteristic of *P. gingivalis* OMVs. Our data positioned PPAD as the central mediator of vascular endothelial glycocalyx injury, thereby expanding the evidence base for PPAD-driven pathological mechanisms in CVDs progression.

The nucleocytoplasmic transport of proteins necessitates the assistance of a complex structure, specifically the nuclear pore complex (NPC) [68]. NPC is a macromolecular structure featuring a central transport channel with a diameter of approximately 9 nm, embedded within the bilayer membrane of the cell nucleus. This channel allows the passage of ions, small molecules, and certain proteins weighing less than 40 kDa, which undergo passive transport for free diffusion. However, molecules with a diameter exceeding 6 nm (≥ 50 kDa) require the assistance of specific carrier proteins for diffusion, specifically karyopherins [69]. The molecular weight of PPAD produced by *P. gingivalis* W83 is ≥ 47kDa [63], and there is currently no literature reporting on whether PPAD can enter the nucleus. Currently, research has demonstrated that PPAD can citrullinate histone H3 in neutrophils [62], yet the mechanism by which PPAD enters the nucleus remains unclear. The present study found that *P. gingivalis* OMVs can induce citrullination of histone H3 in EA. hy926 cells. To further investigate whether PPAD could enter the nucleus and its potential functions, we transfected the overexpressed PPAD plasmid carrying a His-Tag into *P. gingivalis* W83 using bacterial electroporation, resulting in a strain expressing PPAD-His-Tag. Subsequently, through IF colocalization and co-immunoprecipitation experiments, we successfully demonstrated that PPAD could enter the nucleus and interact with histone H3. Through transcription factor prediction and molecular docking simulations, we identified KCNIP3 as a transcriptional repressor of B3GAT1, with histone H3 binding to the transcriptional regulatory region of B3GAT1. CoIP assays in EA. hy926 cells stimulated with OMVs revealed that PPAD induced histone H3 citrullination, enabling CitH3 to recruit KCNIP3, which suppresses B3GAT1 expression; thus, playing a crucial role in glycocalyx injury. However, the method and specific mechanism by which PPAD enters the nucleus merit further investigation in the future.

## Conclusion

In summary, our study revealed elevated levels of serum glycocalyx injury markers in patients with stage III-IV periodontitis. In vitro and in vivo experiments demonstrated that *P. gingivalis* OMVs could induce vascular endothelial glycocalyx injury, with B3GAT1 playing a pivotal role in this process. Our research also preliminarily confirmed the mechanism by which *P. gingivalis* OMVs promoted vascular endothelial glycocalyx injury via the PPAD/CitH3/B3GAT1 pathway, highlighting B3GAT1 as a promising therapeutic target for vascular endothelial glycocalyx injury.

## Supplementary Information

Supplementary Material 1

## Data Availability

All data needed to evaluate the conclusions in the paper are present in the paper and/or the Supplementary Materials. Other data supporting the findings of this study are available from the corresponding author upon reasonable request.

## Abbreviations

AS: Atherosclerosis
AUC: Area under the curve
B3GAT1: β 1,3-glucuronosyltransferase
BOP: Bleeding on probing
CAL: Clinical attachment loss
CitH3: Citrullinated histone H3
CVD: Cardiovascular disease
GAG: Glycosaminoglycan
GPC: Glypican
HA: Hyaluronic acid
HS: Heparan sulphate
IF: Immunofluorescence
KCNIP3: Kv channel interacting protein 3
NO: Nitric oxide
NPC: Nuclear pore complex
NTA: Nanoparticle tracking analysis
OMVs: Outer membrane vesicles
PAD: Peptidylarginine deiminase
*P. gingivalis*: *Porphyromonas gingivalis*
PG: Proteoglycans
PPAD: *Porphyromonas gingivalis* peptidylarginine deiminase
PPD: Probing pocket depth
ROC: Receiver operating characteristic
SDC1: Syndecan-1
TEM: Transmission electron microscope
VEC: Vascular endothelial cell
WB: Western blotting
WGA: Wheat germ agglutinin
