# Supplementary material for: Porphyromonas gingivalis-derived outer membrane vesicles promote vascular endothelial glycocalyx injury via the PPAD/CitH3/B3GAT1 pathway
Source: J Nanobiotechnology. 2026 Jan 9;24:137. doi: 10.1186/s12951-025-04015-4 (PMC12882632; doi:10.1186/s12951-025-04015-4)
Supplement: Supplementary file 1 — Supplementary Material 1 [file 12951_2025_4015_MOESM1_ESM.docx]

**Supplementary Materials**

***Porphyromonas gingivalis*-derived outer membrane vesicles promote vascular endothelial glycocalyx injury via the PPAD/CitH3/B3GAT1 pathway**

**1. Supplementary Figures**

Figure S1. Identification of *P. gingivalis* W83 OMVs.

Figure S2. Schematic diagram of mouse aorta anatomy.

Figure S3. Supplementary materials of mRNA high-throughput sequencing.

Figure S4. KEGG pathway enrichment analysis

Figure S5. RT-qPCR of *B3GAT1*, *BCL2*, *ESR2*, *IRF9*, *PRLR* in EA. hy926 cells treated with *P. gingivalis* OMVs.

Figure S6. Agarose gel electrophoresis of plasmids for B3GAT1 (OE).

Figure S7. Transfection efficiency of siRNA.

Figure S8. Transfection efficiency of the B3GAT1 overexpression plasmid.

Figure S9. Transfection efficiency of the B3GAT1overexpression AAV.

Figure S10. Identification of *P. gingivalis* ΔPG1424 ^ΔPPAD^OMVs.

Figure S11. Agarose gel electrophoresis of DNA for identification of *P. gingivalis* PG1424^OE^.

Figure S12. Molecular simulation of computer docking between PPAD and histone H3.

Figure S13. LC-MS/MS base peak of CoIP with anti-His-Tag.

Figure S14. Prediction of transcriptional regulators of B3GAT1 using ChEA3.

Figure S15. Molecular simulation of computer docking of histone H3, KCNIP3 with B3GAT1 nucleic acid sequence.

Figure S16. Expression changes of SDC1 and HS in EA. hy926 cells treated with *P. gingivalis*-LPS, gingipains, and OMVs.

Figure S17. Immunofluorescence changes of glycocalyx (WGA) in EA. hy926 cells treated with *P. gingivalis*-LPS, gingipains, and OMVs.

Figure S18. Expression changes of SDC1, GPC1 and HS in EA. hy926 cells treated with *P. endodontalis* OMVs, and *P. gingivalis* OMVs.

**2. Supplementary Tables**

Table S1. HR (95% CI) for periodontitis according to serum HS and SDC1 levels.

Table S2. Primer pairs used in the study.

Table S3. Interaction modes, amino acid names and location of Histone H3 and KCNIP3 protein.

Table S4. Interaction modes, amino acid names and location of Histone H3, KCNIP3 protein, and the B3GAT1 nucleic acid sequence.

**3. Supplementary information about gene sequences**

(1) Sequences of siRNA.

(2) Gene sequences of the B3GAT1 overexpression plasmid.

(3) Gene sequences of the B3gat1 overexpression AAV.

**4. Major Resources Table**

**1. Supplementary figures**

**Figure S1**


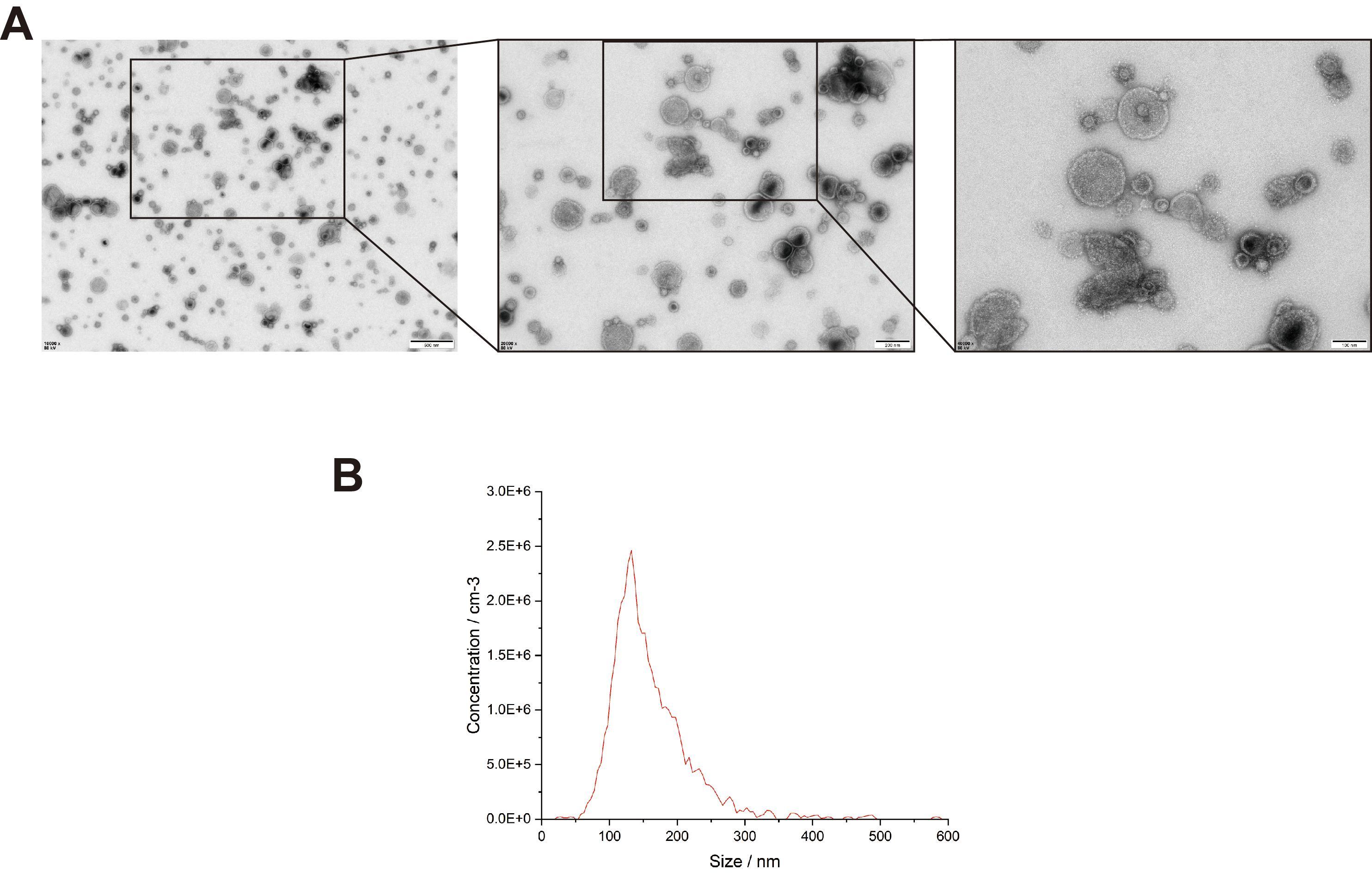


**Figure S1. Identification of *P. gingivalis* W83 OMVs**

A. TEM showed that the OMVs were in the shape of circular vesicles (10000X, 20000X, 40000X); B. NTA showed that the average diameter of the OMVs was approximately 129.8 nm.

**Figure S2**


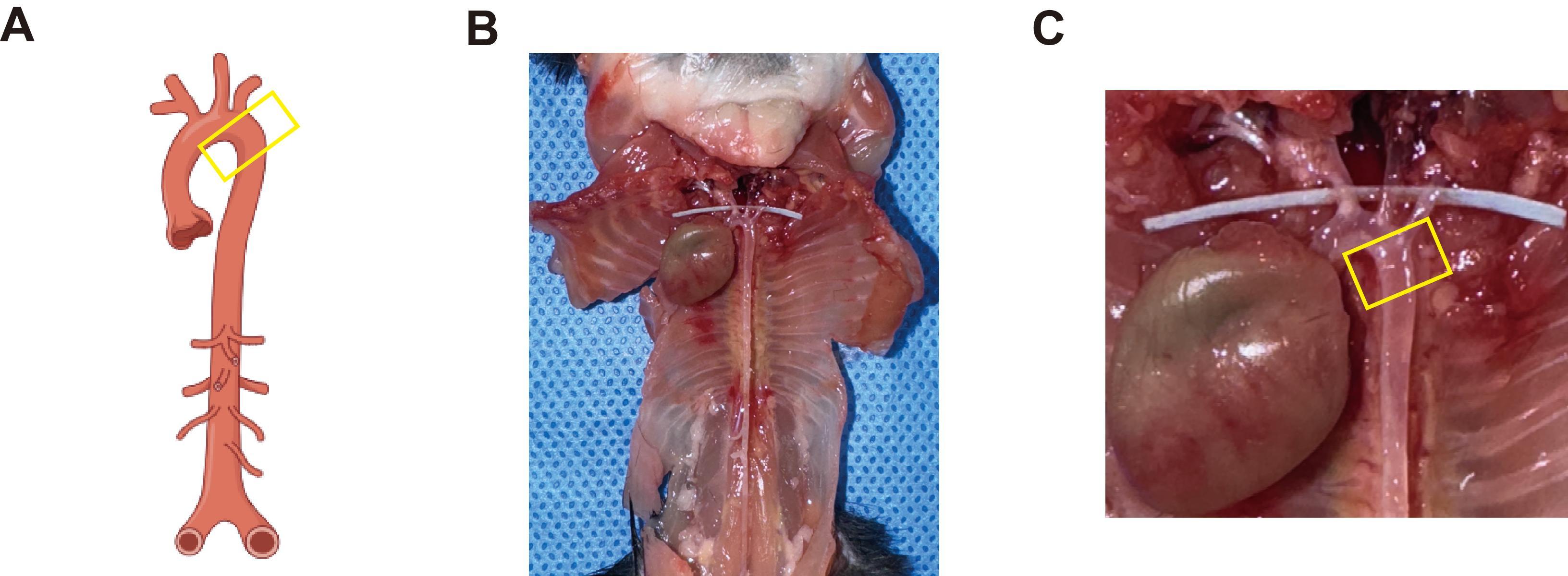


**Figure S2. Schematic diagram of mouse aorta anatomy.**

A. Anatomical diagram of aorta; B, C. Aorta anatomy of C57B/6J mice; The yellow border indicated the location where the tissue sections were cut.

**Figure S3**


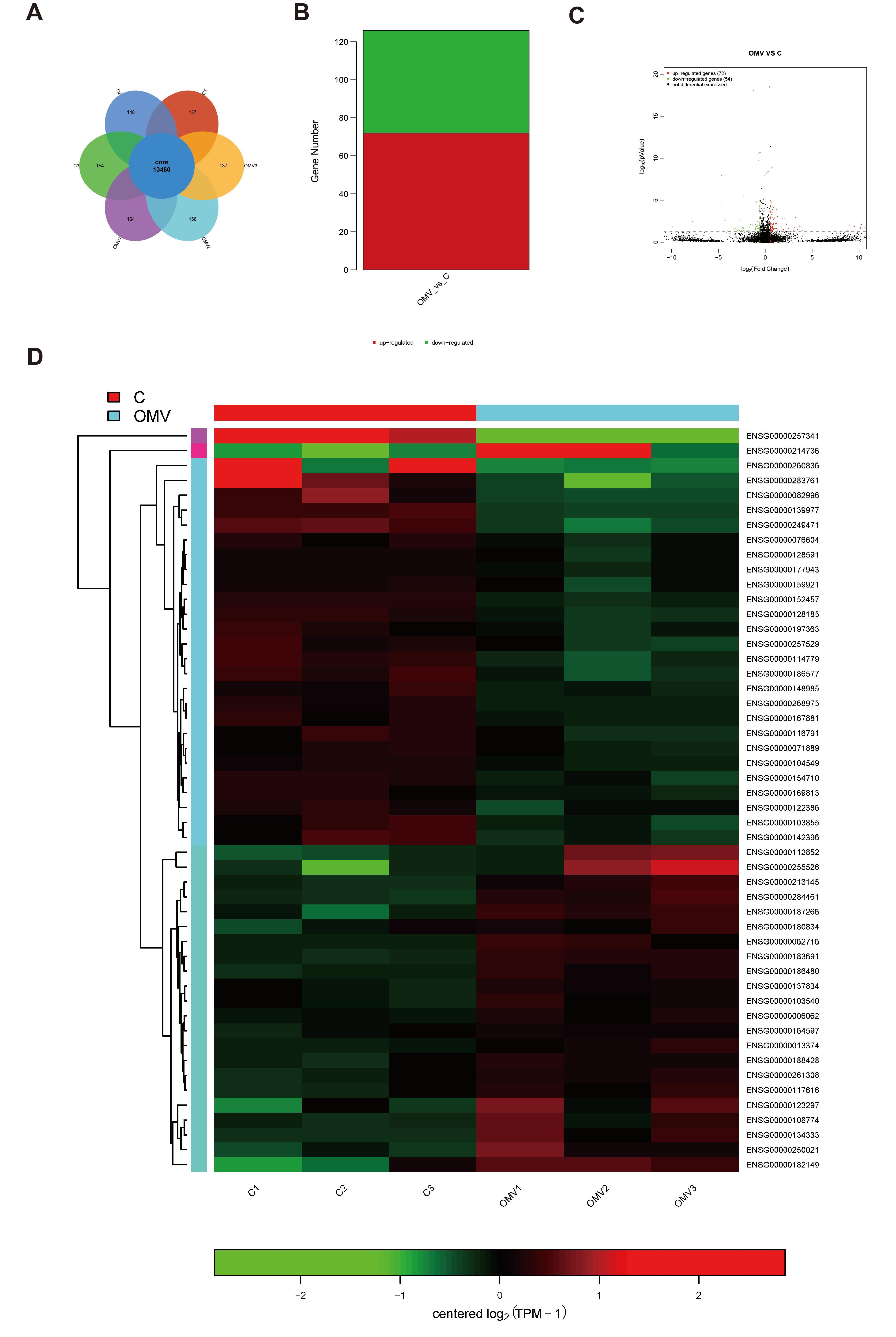


**Figure S3. Supplementary materials of mRNA high-throughput sequencing.**

A. Venn diagram of co-expressed genes, a total of 13, 460 core genes shared among samples; B. Box plot of differentially expressed genes (red indicated upregulated; green indicated downregulated), including 72 upregulated genes and 54 downregulated genes; C. Volcano plot of differentially expressed genes; D. Heat map of top 50 differentially expressed genes, columns representing individual genes and rows corresponding to samples. Red coloration indicated elevated expression levels of specific genes within corresponding samples, while green indicated reduced expression.

**Figure S4**


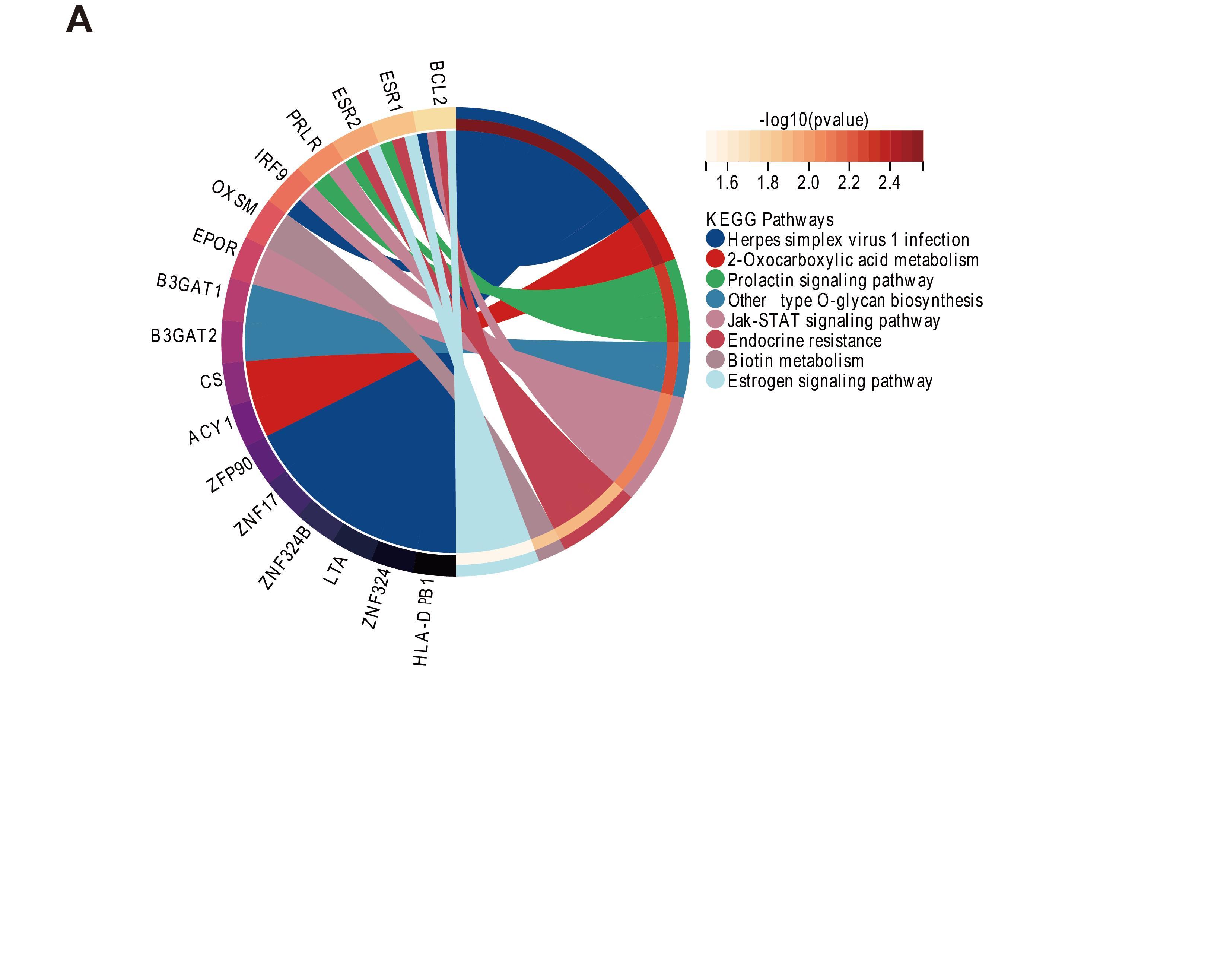


**Figure S4 KEGG pathway enrichment analysis**

A. KEGG pathway enrichment analysis of differentially expressed genes, including other types of O−glycan biosynthesis, glycosaminoglycan degradation, JAK-STAT signaling pathway, cell adhesion molecules and others.

**Figure S5**

**
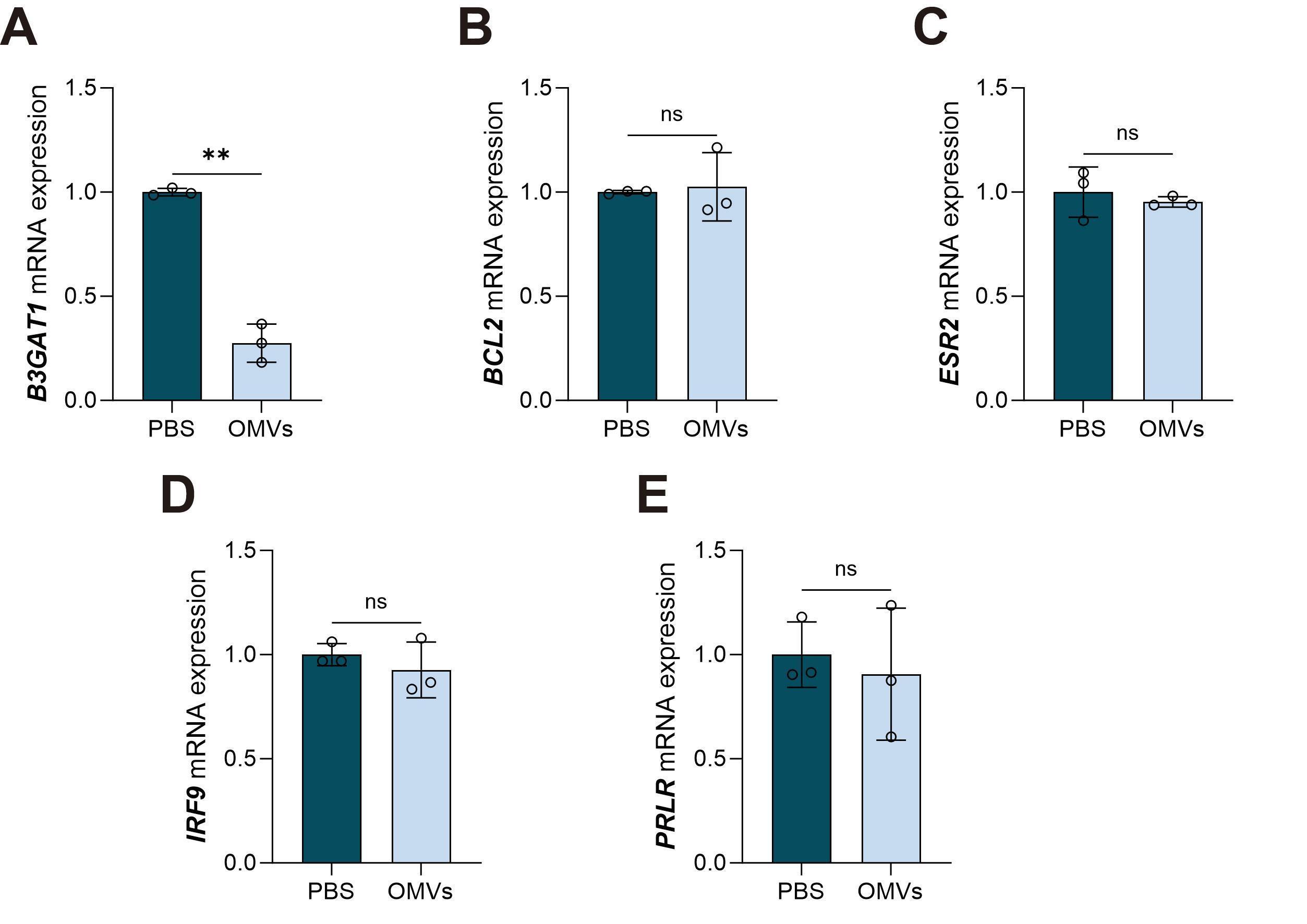
**

**Figure S5 RT-qPCR of *B3GAT1*, *BCL2*, *ESR2*, *IRF9*, *PRLR* in EA. hy926 cells treated with *P. gingivalis* OMVs**

A. RT-qPCR showed that the expression level of *B3GAT1* mRNA in the OMVs group was significantly lower than that in the PBS group. (n = 3, ns *P* > 0.05, ***P* < 0.01)

**Figure S6**

**
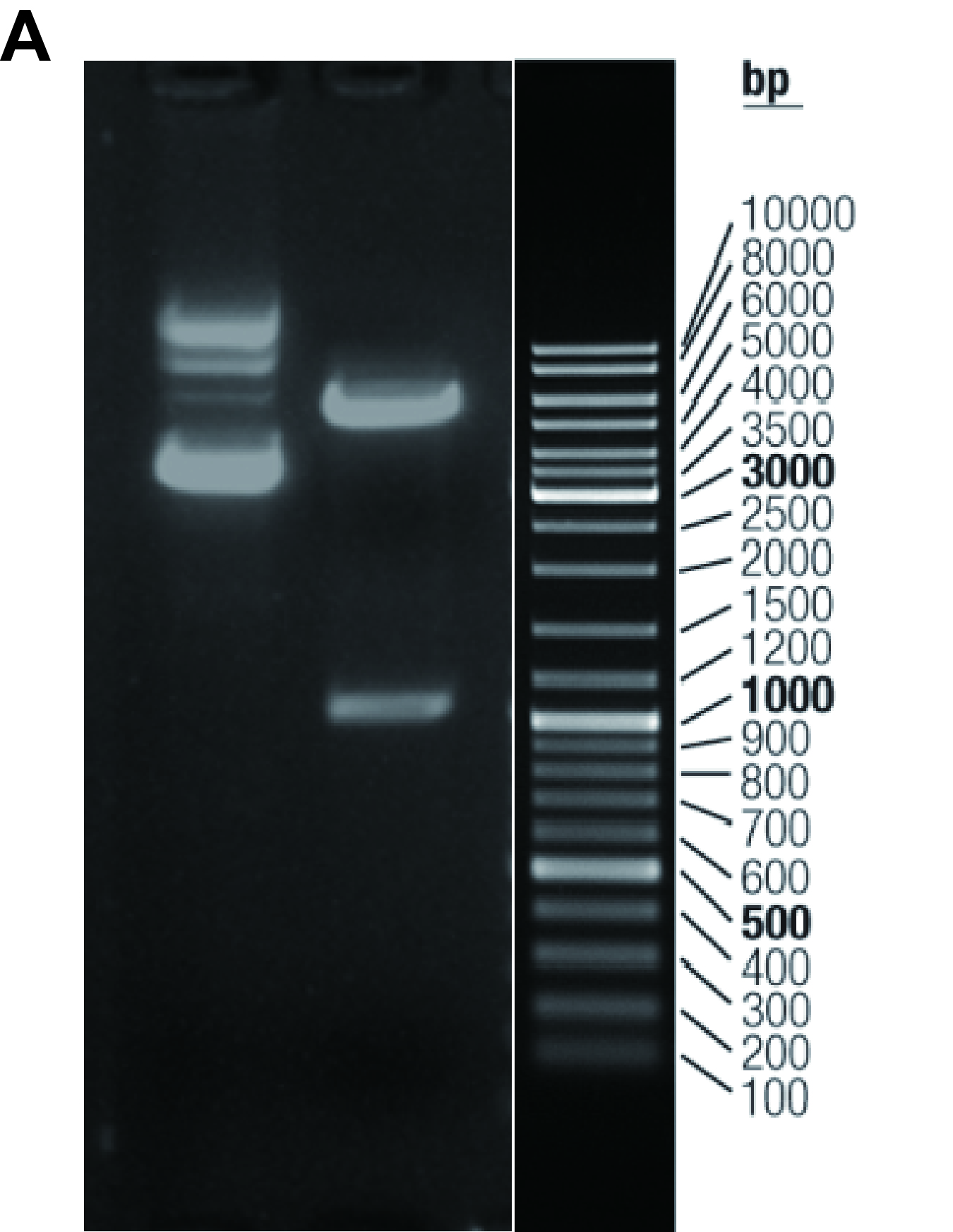
**

**Figure S6. Agarose gel electrophoresis of plasmids for B3GAT1 (OE).**

A. Results of plasmid enzyme digestion. Lane M: KB Ladder; Lane 1: pcDNA3.1(+)-B3GAT1 plasmid; Lane 2: pcDNA3.1(+)-B3GAT1 plasmid digested by SacI and XhoI.

**Figure S7**

**
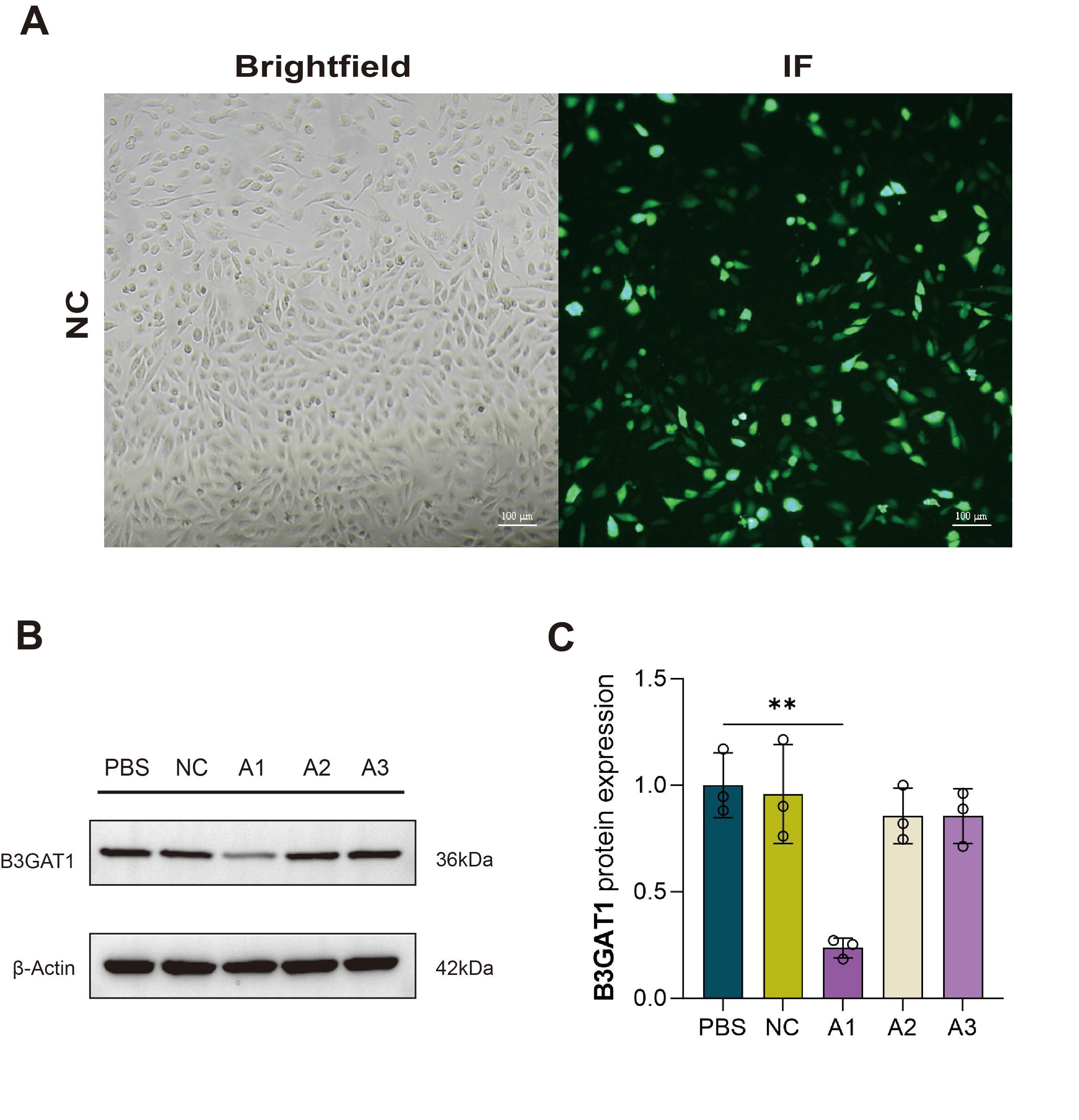
**

**Figure S7 Transfection efficiency of siRNA**

A. Immunofluorescence staining showing the successful transfection of siRNA; B. Immunoblots of B3GAT1 in EA. hy926 cells treated with siRNA; C. Quantitative analysis of B3GAT1 in EA. hy926 cells treated with siRNA; siRNA A1 significantly reduced B3GAT1 protein expression to less than 25%. (n = 3, ***P* < 0.01)

**Figure S8**


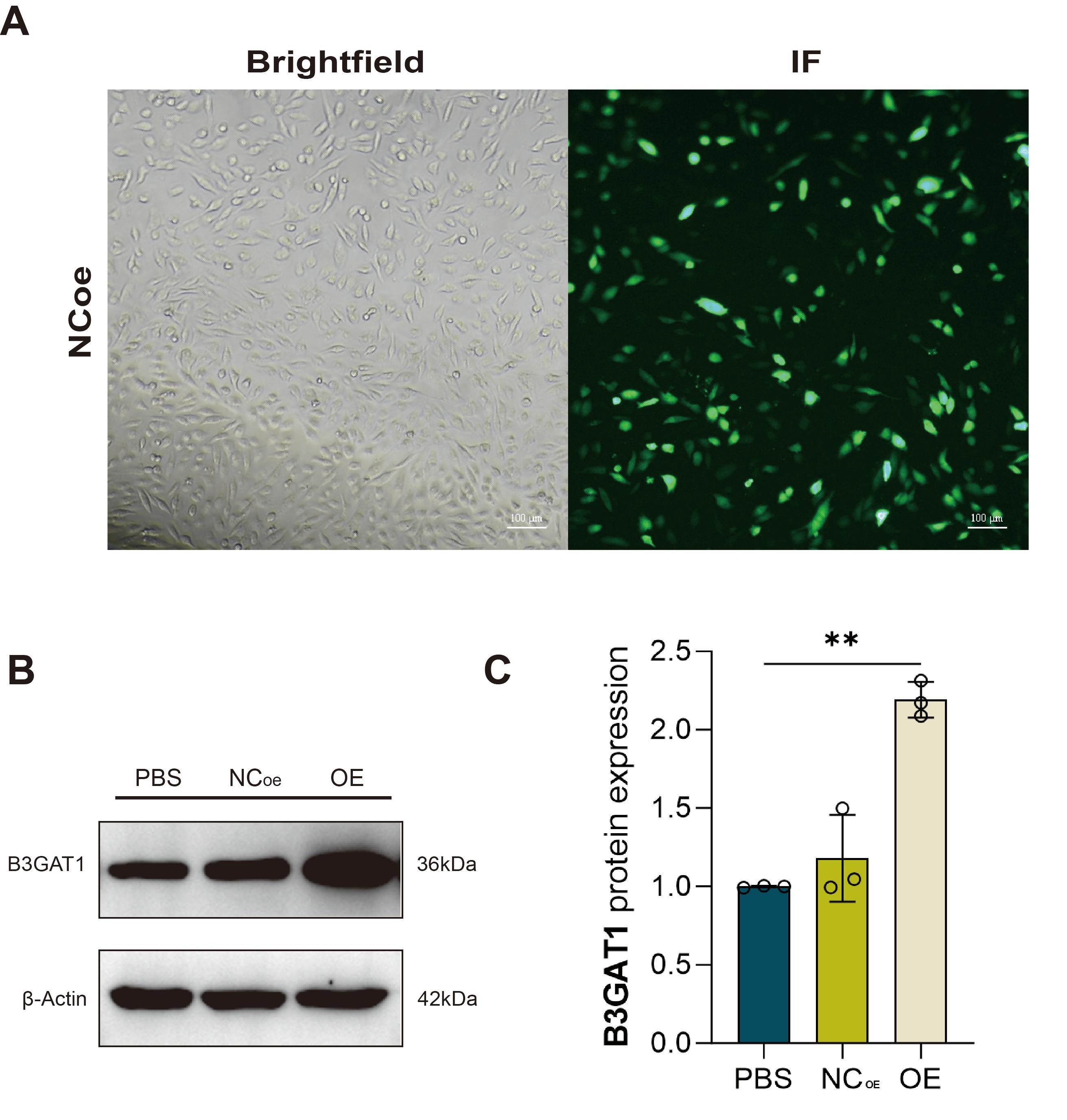


**Figure S8. Transfection efficiency of the B3GAT1 overexpression plasmid.**

A. Immunofluorescence staining showing the successful transfection of overexpressed plasmid; B. Immunoblots of B3GAT1 in EA. hy926 cells treated with the B3GAT1 overexpressed plasmid; C. Quantitative analysis of B3GAT1 in EA. hy926 cells treated with the B3GAT1 overexpressed plasmid, the OE plasmid significantly increased B3GAT1 protein expression by over 2.2 times. (n = 3, ***P* < 0.01)

**Figure S9**

**
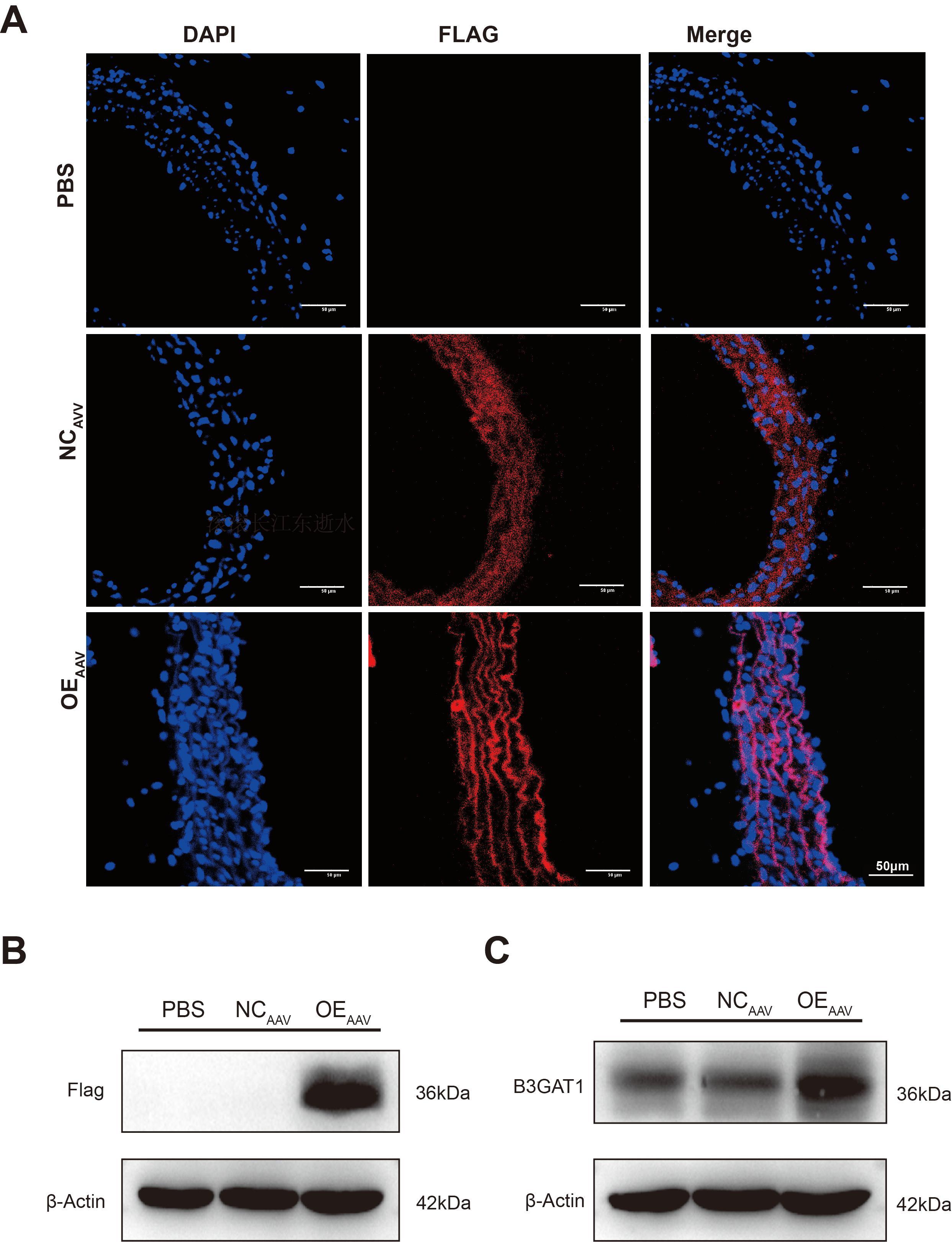
**

**Figure S9 Transfection efficiency of the B3GAT1overexpression AAV.**

A. Immunofluorescence staining showing the FLAG expression in the mouse aorta after tail vein injection of AAV; B, C. Immunoblots of FLAG and B3GAT1 in the mouse aorta after tail vein injection of AAV, WB test showed positive Flag expression and significantly increased B3GAT1 protein expression in the OE_AAV_ group.

**Figure S10**


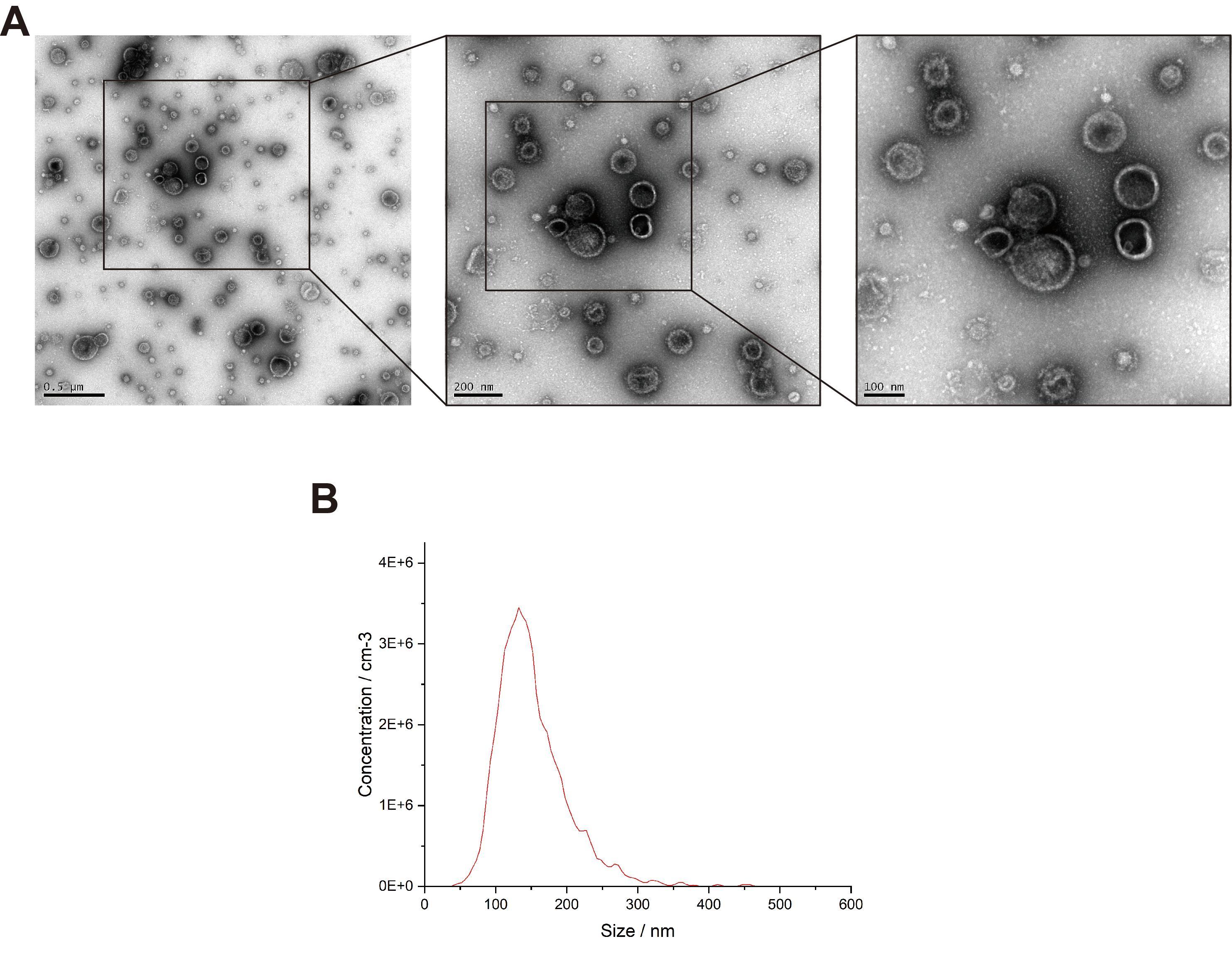


**Figure S10** **Identification of *P. gingivalis* ΔPG1424 ^ΔPPAD^OMVs.**

A. TEM showed that the ^ΔPPAD^OMVs were also in the shape of circular vesicles (30000X, 60000X, 100000X); B. NTA showed that the average diameter of the OMVs was approximately 133.9 nm.

**Figure S11**


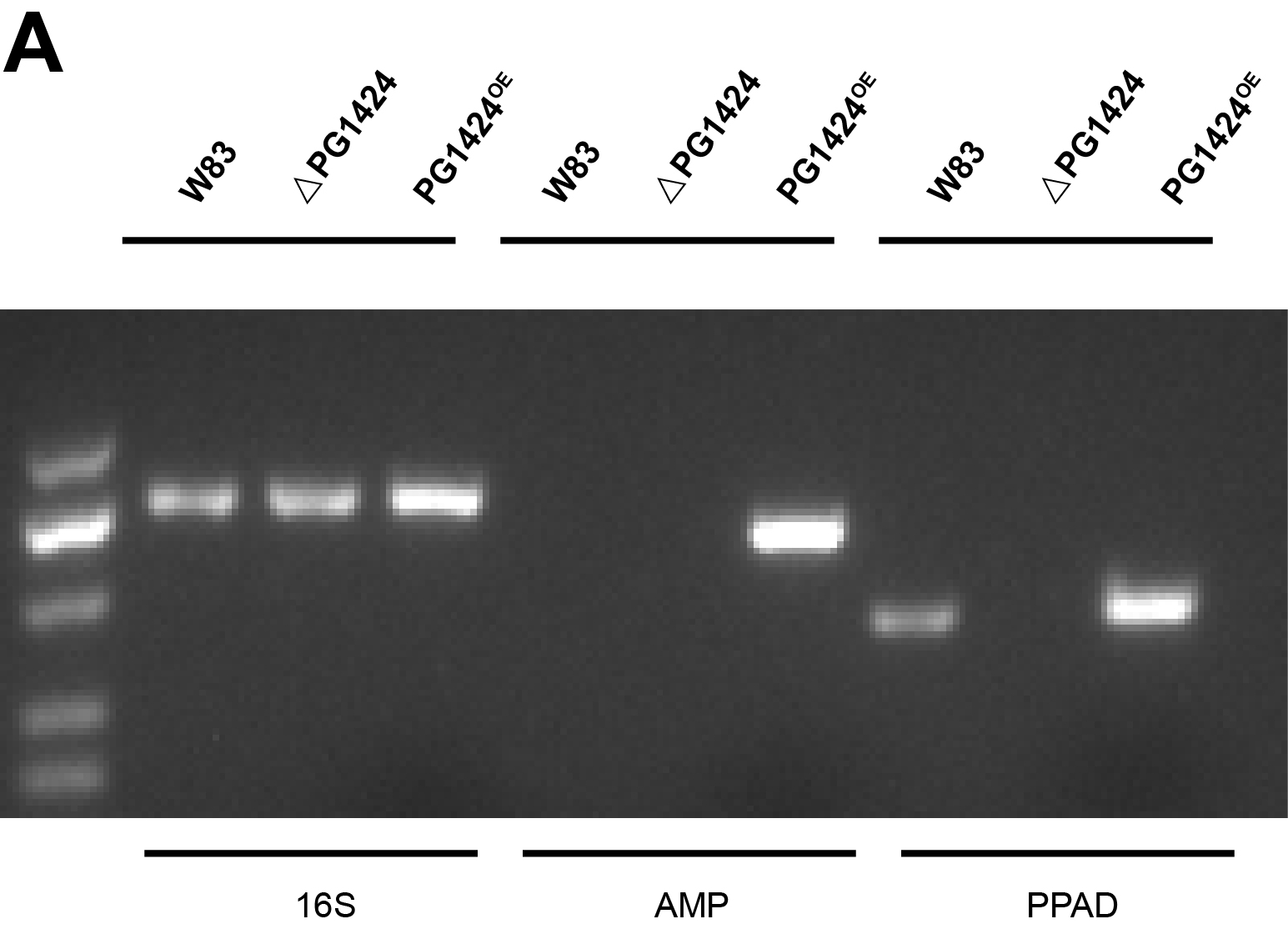


**Figure S11 Agarose gel electrophoresis of DNA for identification of** ***P. gingivalis* PG1424^OE^**

A. *P. gingivalis* W83, *P. gingivalis* ΔPG1424 and *P. gingivalis* PG1424^OE^ all expressed 16S. Since the PG1424^OE^ strain contained an ampicillin (AMP) resistance gene introduced by electroporation, only *P. gingivalis* PG1424^OE^expressed AMP, while *P. gingivalis* ΔPG1424 did not express PPAD. The above results showed the reliability of the three strains used in our experiments.

**Figure S12**

**
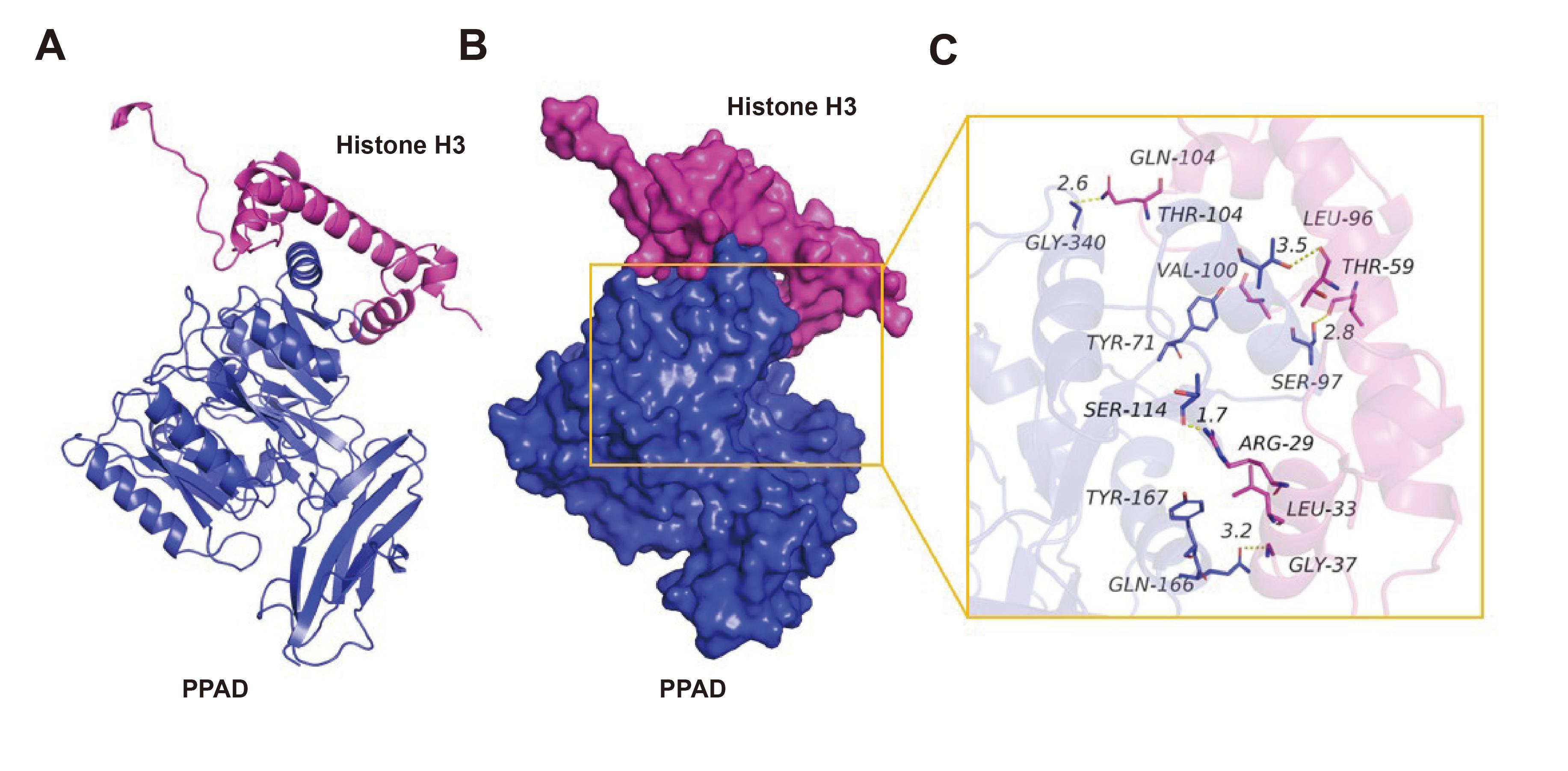
**

**Figure S12** **Molecular simulation of computer docking between PPAD and histone H3.**

A. Surface representation of PPAD and histone H3; B. Tube representation of the protein backbones, colored blue (PPAD) and red (H3); C. Detailed binding mode between PPAD and H3. Yellow dashed lines represent hydrogen bonds.

**Figure S13**

**
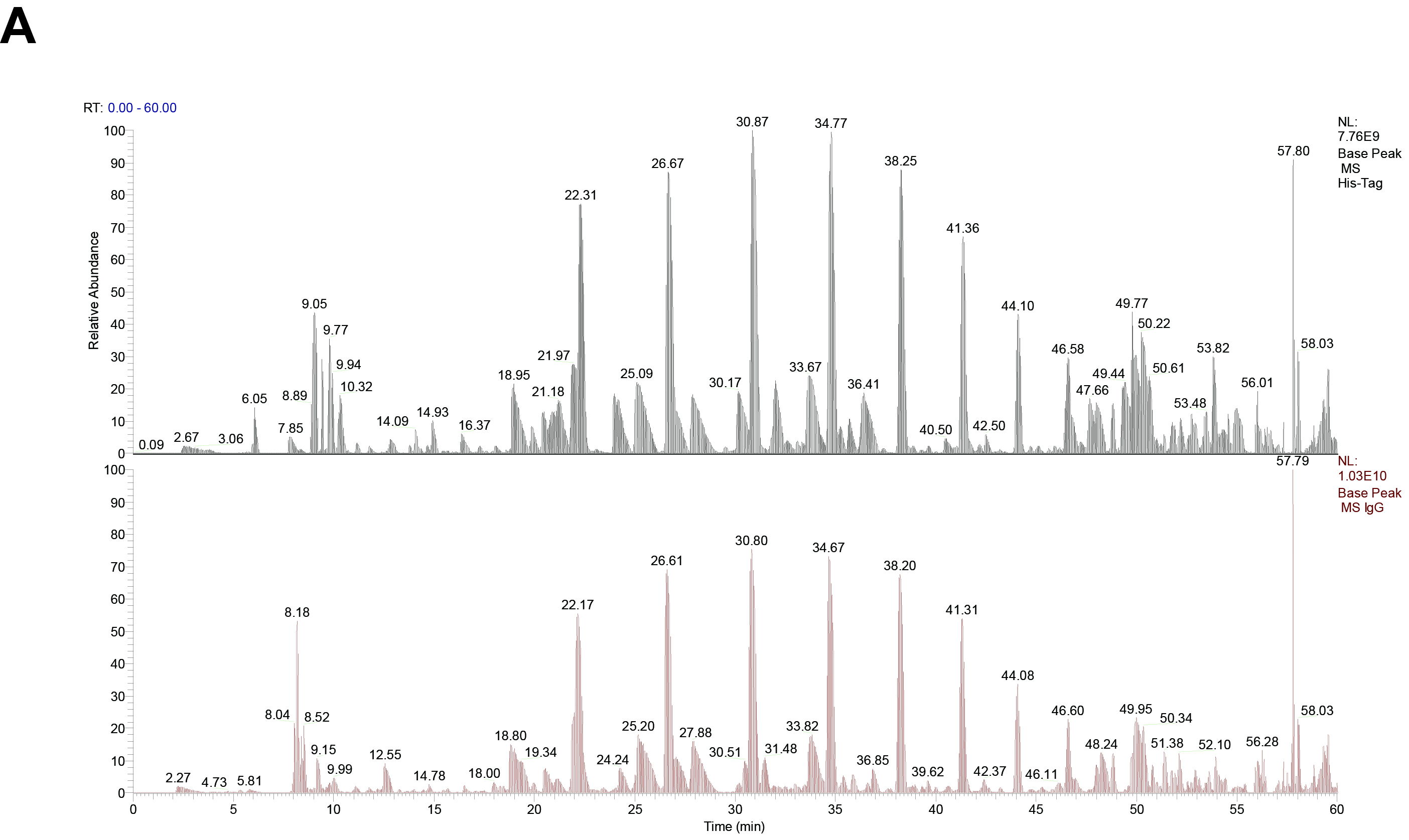
**

**Figure S13** **LC-MS/MS base peak of CoIP with anti-His-Tag.**

A. The LC-MS detected a total of 222 proteins in the His-Tag group samples and 250 proteins were detected in the IgG group samples. Histone H3 was detected in both sample groups; The iBAQ values were 9,918,900 for the His-Tag group and 416,220 for the IgG group.

**Figure S14**

**
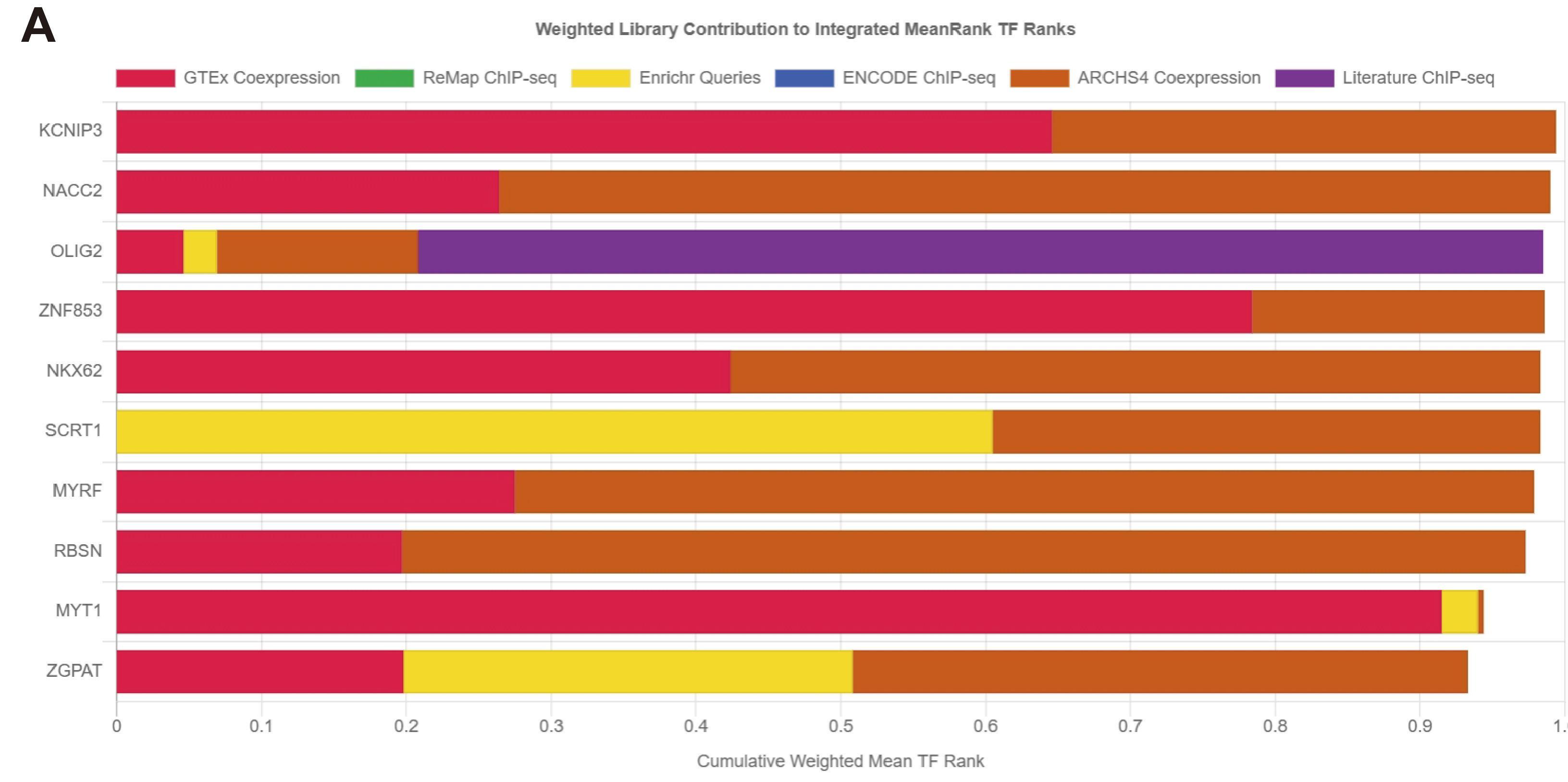
**

**Figure S14** **Prediction of transcriptional regulators of B3GAT1 using ChEA3.**

A. ChEA3 database analysis identified potential transcriptional regulators of B3GAT1. The top 10 candidates ranked by Mean Rank scores as follows: KCNIP3 (10.0), NACC2, OLIG2, ZNF853, NKX62, SCRT1, MYRF, RBSN, MYT1, and ZGPAT. Lower Mean Rank values indicated stronger consensus across multiple databases and higher predictive confidence for transcription factor associations.

**Figure S15**

**
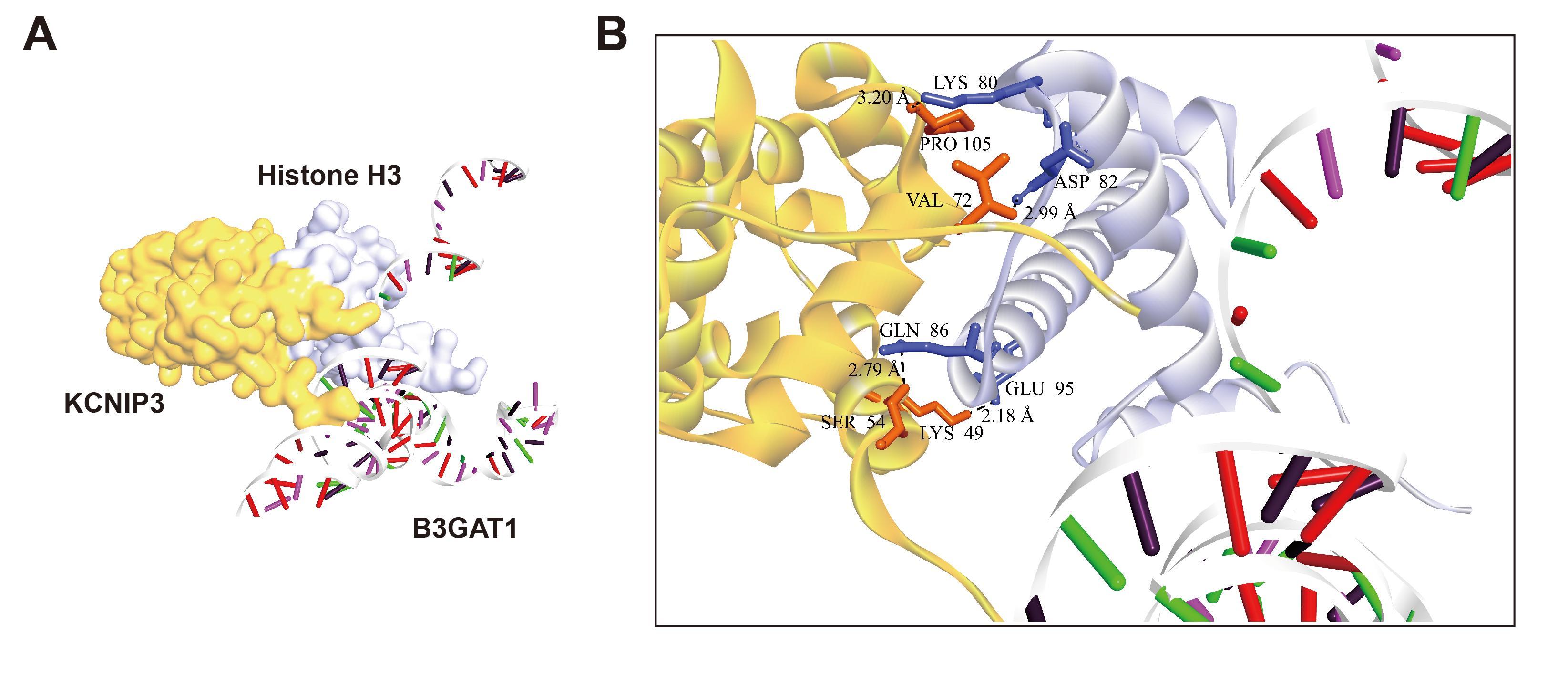
**

**Figure S15** **Molecular simulation of computer docking of histone H3, KCNIP3 with B3GAT1 nucleic acid sequence.**

A. Global view, Histone H3 protein in purple, KCNIP3 protein in yellow, and B3GAT1 transcriptional regulatory region in other parts; B. Map of specific sites of interaction.

**Figure S16**

**
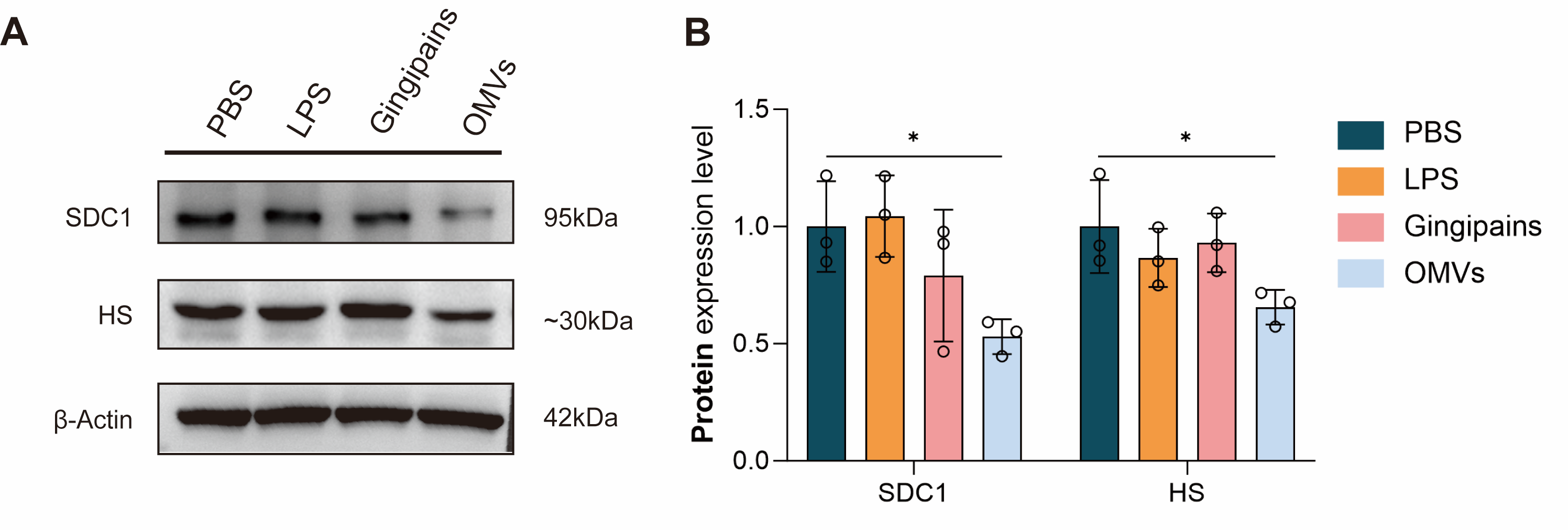
**

**Figure S16** **Expression changes of SDC1 and HS in EA. hy926 cells treated with *P. gingivalis*-LPS, gingipains, and OMVs**

A. Immunoblots of SDC1 and HS in EA. hy926 cells treated with *P. gingivalis*-LPS, gingipains, and OMVs, the expression of SDC1 and HS decreasing significantly in the OMVs group; B. Quantitative analysis of the expression for SDC1 and HS. (n = 3, **P* < 0.05)

**Figure S17**

**
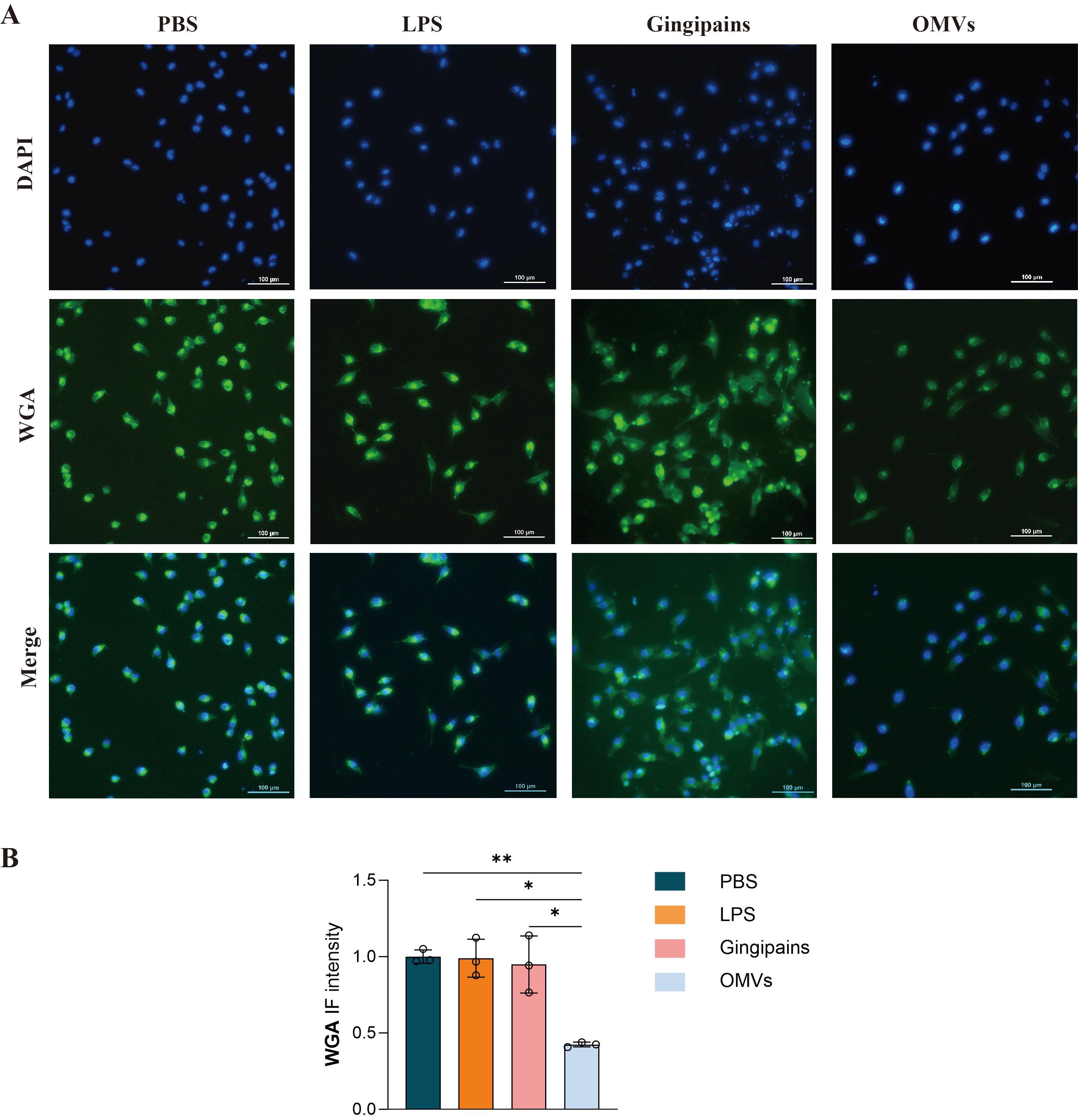
**

**Figure S17** **Immunofluorescence changes of glycocalyx (WGA) in EA. hy926 cells treated with** ***P. gingivalis*-LPS, gingipains, and OMVs.**

A. Immunofluorescence staining showing changes in glycocalyx (WGA) of EA. hy926 cells after stimulation with *P. gingivalis*-LPS, gingipains, and OMVs, fluorescence intensity decreasing significantly in the OMVs group; B. Relative fluorescent intensity (density). (n = 3, **P* < 0.05, ***P* < 0.01)

**Figure S18**

**
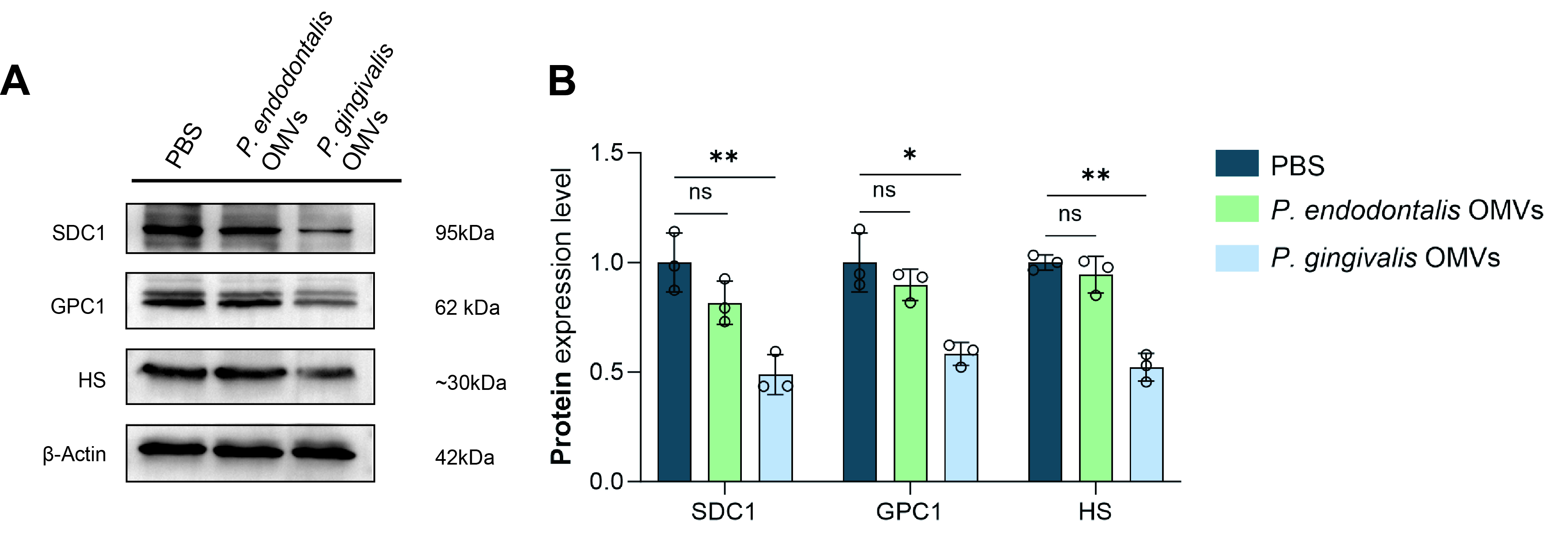
**

**Figure S18** **Expression changes of SDC1, GPC1and HS in EA. hy926 cells** **treated with** ***P. endodontalis* OMVs and *P. gingivalis* OMVs**

A. Immunoblots of SDC1, GPC1 and HS in EA. hy926 cells treated with treated with *P. endodontalis* OMV*s* and *P. gingivalis* OMVs, the expression of SDC1, GPC1 and HS decreasing significantly in the *P. gingivalis* OMVs group; B. Quantitative analysis of the expression for SDC1, GPC1 and HS. (n = 3, ns *P* > 0.05, **P* < 0.05, ***P* < 0.01)

**2. Supplementary tables**

Table S1. HR (95% CI) for periodontitis according to HS and SDC1.

| Outcome | Periodontal status | | *P* value |
| --- | --- | --- | --- |
|  | NO/Stages I–II Periodontitis | Stages III–IV Periodontitis |  |
| HS |  |  |  |
| Model 0^a^ | 1.00 | 7.91 (2.84, 22.05) | < 0.001 |
| Model 1^b^ | 1.00 | 8.91 (1.91, 41.49) | 0.005 |
| Model 2^c^ | 1.00 | 8.72 (1.21, 63.05) | 0.032 |
| Model 3^d^ | 1.00 | 9.50 (1.19, 75.98) | 0.034 |
| SDC1 |  |  |  |
| Model 0 ^a^ | 1.00 | 4.22 (1.61, 11.04) | 0.003 |
| Model 1 ^b^ | 1.00 | 5.71 (1.29, 25.34) | 0.022 |
| Model 2 ^c^ | 1.00 | 5.35 (0.72, 40.02) | 0.102 |
| Model 3^d^ | 1.00 | 5.34 (0.66, 43.20) | 0.116 |

*Abbreviations*: HR, hazard ratio; CI, confidence interval; HS, heparan sulfate; SDC1, syndecan-1.

Median of HS: 0.3837 ng/mL; Median of SDC1: 6.6562 ng/mL.

^a^ Model 0 was univariate analysis.

^b^ Model 1 included age, sex (male or female).

^c^ Model 2, based on Model 1, we further adjusted for smoking status (never smokers, former, or current smokers), drinking status (non-drinker, light drinker, moderate drinker).

^d^ Model 3, based on Model 2, we further adjusted for systemic diseases associated with periodontitis including hypertension status (no, or yes) and diabetes status (no, or yes).

Table S2. Primer pairs designed in the study.

| Gene name/gene ID | Primer pairs |
| --- | --- |
| *β-Actin*  (NM_001101.5) | F: GACAGTCAGCCGCATCTTCT  R: GCGCCCAATACGACCAAATC |
| *B3GAT1*  (NM_001367973.1) | F: GAAAGCAGCCTCCTTCGAGAAC  R: CCTCATTCACCAGCACTGGCTT |
| *BCL2*  (NM_000633.3) | F: ATGTGTGTGGAGAGCGTCAACC  R: TGAGCAGAGTCTTCAGAGACAGCC |
| *ESR2*  (NM_001040275.1) | F: CTGATGCGACAGCTCTGA  R: TGTATGTTCGGTCCAAGTC |
| *IRF9*  (NM_001385400.1) | F: TGGCATCAGGCAGGGCACGCTG  R: GAACTGTGCTGTCGCTTTGATGG |
| *PRLR*  (NM_000949.7) | F: ATTCTCCTCCATTACCTT  R: CTGGGATTACCATTGTTG |
| *PPAD*  (WP_005873463.1) | F: ATGAAAAAGCTTTTACAGGCTAAAGCCTTG  R: TTATTTGAGAATTTTCATTGTCTCACGGATTCC |
| *AMP*  (NP_308142.1) | F: FGGTCGCCGCATACACTATTCTCAG  R: GCAACTTTATCCGCCTCCATCCAG |
| *16sRNA*  (NC_069041.1) | F: CATGTACGTTGCTATCCAGGC  R: CTCCTTAATGTCACGCACGAT |

Table S3. Interaction mode, amino acid name and location of Histone H3 and KCNIP3 protein.

| Protein1 | Protein2 | Binding Energy (kcal/mol) | Contact Sites (PPAD) | Contact Sites (H3) | Combination  Type |
| --- | --- | --- | --- | --- | --- |
| PPAD | H3 | -252.53 | TYR-71, TYR-167, SER-97, THR-104, GLN-166, SER-114, GLY-340 | VAL-100, LEU-33, LEU-96, THR-59, GLY-37, ARG-29, GLN-104 | Hydrogen bond,  Hydrophobic interaction |

Table S4. Interaction mode, amino acid name and location of Histone H3, KCNIP3 protein and B3GAT1 nucleic acid sequence.

| Histone H3 | B3GAT1_TF | Distance [Å] | Categoty | Types |
| --- | --- | --- | --- | --- |
| ARG54 | C47 | 3.6158 | Hydrogen Bond;  Electrostatic | Salt Bridge |
| ARG70 | U163 | 2.27666 | Hydrogen Bond;  Electrostatic | Salt Bridge |
| ARG50 | C13 | 5.45684 | Electrostatic | Attractive Charge |
| ARG53 | C12 | 5.12192 | Electrostatic | Attractive Charge |
| ARG70 | A164 | 5.08632 | Electrostatic | Attractive Charge |
| TYR42 | C13 | 3.30336 | Hydrogen Bond | Conventional Hydrogen Bond |
| ARG54 | G46 | 3.22878 | Hydrogen Bond | Conventional Hydrogen Bond |
| TYR55 | G168 | 3.38502 | Hydrogen Bond | Conventional Hydrogen Bond |
| Continued | | | | |
| Histone H3.3 | KCNIP3 | Distance [Å] | Types | |
| LYS  80 | PRO 105 | 3.20 | Hydrogen bonds | |
| ASP  82 | VAL  72 | 2.99 | Hydrogen bonds | |
| GLN  86 | SER  54 | 2.79 | Hydrogen bonds | |
| GLU  95 | LYS  49 | 2.18 | Hydrogen bonds and Salt bridges | |

**3. Supplementary information about gene sequences**

**(1) Sequences of siRNA.**

| **Lot No** | **Oligo Name** | **Sequences (5' to 3')** | **Length** |
| --- | --- | --- | --- |
| A1 | hB3GAT1-2868-s | /ru//ru//rg//rg//ra//ra//ra//ru//ru//ra//ru//ru//rc//ru//ra//ra/ /ra//rc//ra/tt | 21 |
| A1 | hB3GAT1-2868-a | /ru//rg//ru//ru//ru//ra//rg//ra//ra//ru//ra//ra//ru//ru//ru//rc/ /rc//ra//ra/tt | 21 |
| A2 | hB3GAT1-3594-s | /rc//ra//rg//rg//ra//rc//ru//ru//ru//rg//ra//ru//ra//ra//ru//rg/ /ra//ra//rc/tt | 21 |
| A2 | hB3GAT1-3594-a | /rg//ru//ru//rc//ra//ru//ru//ra//ru//rc//ra//ra//ra//rg//ru//rc/ /rc//ru//rg/tt | 21 |
| A3 | hB3GAT1-3740-s | /ra//ru//rg//ra//rg//ru//ru//ru//rg//ru//ru//ra//ru//ra//ra//ra/ /ru//rc//ra/tt | 21 |
| A3 | hB3GAT1-3740-a | /ru//rg//ra//ru//ru//ru//ra//ru//ra//ra//rc//ra//ra//ra//rc//ru/ /rc//ra//ru/tt | 21 |

**(2) Gene sequences of the B3GAT1 overexpressed plasmid.**

(Based on NM_054025.3)

GCTAGCGCCACCATGCCGAAGAGACGGGACATCCTAGCGATCGTCCTCATCGTGCTGCCCTGGACTCTGCTCATCACTGTCTGGCACCAGAGCACCCTCGCACCCCTGCTCGCGGTACATAAGGATGAGGGCAGTGACCCCCGACGCGAAACGCCGCCCGGCGCCGACCCCAGGGAGTACTGCACGTCTGACCGCGACATCGTGGAGGTGGTGCGCACCGAGTACGTGTACACGCGGCCCCCGCCATGGTCCGACACGCTGCCCACCATCCACGTGGTGACGCCCACCTACAGCCGCCCGGTGCAGAAGGCCGAGCTGACGCGCATGGCCAACACGCTGCTGCACGTGCCCAACCTCCACTGGCTGGTGGTGGAGGATGCGCCGCGCCGGACGCCGCTGACCGCGCGCCTGCTGCGCGACACCGGCCTCAACTACACGCACCTGCACGTGGAGACGCCCCGCAACTACAAGCTGCGCGGAGACGCCCGCGACCCACGCATCCCGCGGGGCACCATGCAGCGCAACCTGGCCCTGCGCTGGCTGCGCGAGACCTTCCCGCGCAACTCCAGCCAGCCTGGCGTGGTCTACTTCGCCGACGACGACAACACCTACAGCCTGGAGCTCTTCGAAGAGATGCGCAGCACCAGGAGGGTGTCCGTGTGGCCCGTCGCCTTCGTGGGTGGCCTGCGGTACGAGGCCCCACGGGTGAACGGGGCAGGGAAGGTGGTCGGCTGGAAGACGGTGTTTGACCCCCACCGGCCATTTGCAATAGACATGGCTGGATTTGCCGTCAACCTGCGGCTCATTCTGCAGCGAAGCCAGGCCTACTTCAAGCTGCGAGGTGTGAAGGGAGGCTACCAGGAAAGCAGCCTCCTTCGAGAACTTGTCACCCTCAACGACCTGGAGCCCAAGGCAGCCAACTGCACCAAGATCCTGGTGTGGCACACACGGACAGAGAAGCCAGTGCTGGTGAATGAGGGCAAGAAGGGCTTCACTGACCCCTCGGTGGAGATCTGAGGATCC

**(3) Gene sequences of the B3gat1 overexpressed AAV.** (Based on NM_029792.1)

GGTGGGAGGTCTATATAAGCAGAGCTGGTTTAGTGAACCGTCAGATCCGCTAGCCGCCACCATGCCGAAGAGACGGGACATCCTTGCGATTGTCCTCATCGTGCTTCCCTGGACACTGCTCATCACCGTCTGGCACCAGAGCAGCCTCGCACCTCTGCTTGCTGTGCACAAGGATGAGGGAAGTGACCCCCGCCATGAGGCACCACCTGGTGCGGACCCTAGGGAGTACTGCATGTCCGACCGTGACATCGTGGAAGTGGTGCGCACAGAGTACGTGTACACGAGGCCGCCACCCTGGTCCGACACGCTGCCCACCATCCATGTGGTGACGCCCACCTACAGTAGACCGGTGCAGAAGGCAGAGCTGACGCGAATGGCCAACACACTACTGCATGTGCCCAACCTTCACTGGCTAGTGGTGGAGGATGCTCCACGCAGGACGCCCCTCACCGCGCGCTTGCTGCGCGACACTGGCCTCAACTACACACACCTGCACGTAGAGACACCACGCAACTATAAGCTGCGAGGTGATGCCCGAGACCCTCGCATCCCACGTGGCACCATGCAGCGCAACCTGGCACTGCGCTGGCTGCGGGAGACCTTCCCACGGAACTCTACTCAGCCAGGCGTAGTGTACTTCGCGGATGATGACAACACGTACAGTCTGGAGCTCTTTGAAGAGATGCGCAGCACAAGAAGGGTGTCCGTGTGGCCTGTGGCCTTTGTTGGTGGCCTTCGGTATGAGGCCCCACGGGTGAATGGGGCAGGGAAAGTGGTTGGCTGGAAGACAGTCTTCGACCCCCACCGGCCCTTTGCAATAGACATGGCTGGATTTGCTGTCAACCTCCGGCTCATCTTGCAGCGAAGTCAAGCCTACTTTAAGCTACGTGGTGTGAAGGGAGGCTACCAGGAAAGCAGTCTCCTTCGAGAACTTGTCACCCTCAATGACCTGGAGCCCAAGGCAGCAAACTGTACCAAGATCTTGGTCTGGCATACACGAACAGAGAAGCCAGTGCTGGTCAATGAGGGGAAGAAGGGCTTCACCGACCCCTCAGTGGAGATCACCGGTGCCACCATGGACTACAAGGATGACGATGACAAGGATTACAAAGACGACGATGATAAGGACTATAAGGATGATGACGACAAATAAGTTTAAACAAGCTTGGTTATCGATAATCAACCTCTGGATTACAAAATTTGTGAAAGATTGACTGGTATTCTTAACTATGTTGCTCCTTTTACGCTATGTGGATACGCTGCTTTAATGCCTTTGTATCATGCTATTGCTTCCCGTATGGCTTTCATTTTCTCCTCCTTGTATAAATCCTGGTTGCTGTCTCTTTATGAGGAGTTGTGGCCCGTTGTCAGGCAACGTGGCGTGGTGTGCACTGTGTTTGCTGACGCAACCCCCACTGGTTGGGGCATTGCCACCACCTGTCAGCTCCTTTCCGGAACTTTCGCTTTCCCCCTCCCTATTGCCACGGCGGAACTCATCGCCGCCTGCCTTGCCCGCTGCTGGACAGGGGCTCGGCTGTTGGGCACTGA

**4. Major Resources Table**

| Resource | | Source | Identifier |
| --- | --- | --- | --- |
| Antibodies | | | |
| Anti-FITC-WGA | | GeneTex | GTX01502 |
| Rat Anti- HS | | Abcam | ab2501 |
| 6*His, His-Tag Monoclonal antibody | | Proteintech | 66005-1-Ig |
| Anti-FLAG M2 antibody | | Cell Signaling Technology | #2368 |
| Goat Anti-Rat IgG (Alexa Fluor® 488) | | Abcam | ab150157 |
| Multi-rAb CoraLite® Plus 594-Goat Anti-Mouse Recombinant Secondary Antibody (H+L) | | Proteintech | RGAM004 |
| Multi-rAb CoraLite® Plus 488-Goat Anti-Rabbit Recombinant Secondary Antibody (H+L) | | Proteintech | RGAR002 |
| Multi-rAb CoraLite® Plus 594-Goat Anti-Rabbit Recombinant Secondary Antibody (H+L) | | Proteintech | RGAR004 |
| Rabbit Anti- SDC1 | | Abclonal | A4174 |
| Rabbit Anti- GPC1 | | Abclonal | A13019 |
| Rabbit Anti- B3GAT1 | | Abclonal | A9871 |
| Rabbit Anti-Histone H3 | | Abcam | ab1791 |
| Rabbit Anti-Histone H3 (citrulline R2 + R8 + R17) | | Abcam | ab281584 |
| Mouse Anti-O-Linked N-Acetylglucosamine [RL2] | | Abcam | ab2739 |
| Rabbit Anti-RL2 | | Abcam | ab93858 |
| HRP-conjugated Rabbit Anti His-Tag mAb | | Abclonal | AE104 |
| HRP-conjugated Beta Actin Monoclonal antibody | | Proteintech | HRP-66009 |
| HRP-conjugated Goat anti-Mouse IgG (H+L) | | Abclonal | AS003 |
| HRP-conjugated Goat anti-Rat IgG (H+L) | | Abclonal | AS014 |
| HRP-conjugated Goat anti-Rat IgG (H+L) | | Abclonal | AS028 |
| Critical commercial assays | | | |
| ELISA Kit for Heparan Sulfate (HS) | | Cloud-Clone Corp. | CEA161Ge |
| ELISA Kit for Syndecan 1 (SDC1) | | Cloud-Clone Corp. | SEB966Hu |
| Cell Counting Kit-8 (CCK-8) | | Abbkine | KTA1020 |
| Nuclear and Cytoplasmic Protein Extraction Kit | | MeilunBio | MA0211 |
| rProtein A/G Magnetic IP/Co-IP Kit | | Absin | abs9649 |
| FITC Phalloidin | | Solarbio | CA1620 |
| Antifade Mounting Medium with DAPI | | Beyotime | P0131 |
| DID (1,1'-Dioctadecyl-3,3,3',3'-Tetramethylindodicarbocyanine,4-Chlorobenzenesulfonate Salt) | | Beyotime | C1039 |
| PrimeScript™ RT reagent Kit with gDNA Eraser | | TaKaRa | RR047A |
| TB Green® Premix Ex Taq™ II | | TaKaRa | RR820A |
| Color PAGE Gel Rapid Preparation Kit | | Epizyme Biotech | PG112/PG113 |
| Experimental models: Cell lines | | | |
| EA. hy926 | | Procell, China | Cat NO.: CL-0272 |
| THP-1 | | Procell, China | Cat NO.: CL-0233 |
| Experimental models: Bacterial | | | |
| Name | Vendor or Source | Background Strain | Other Information |
| *P. gingivalis* W83 | Department of Periodontology, School and Hospital of Stomatology, China Medical University |  |  |
| *P. gingivalis* △PG1424 | Department of Periodontology, School and Hospital of Stomatology, China Medical University | *P. gingivalis* W83 | Knockout of PG1424(PPAD) gene |
| *P. gingivalis* PG1424^OE^ | Department of Periodontology, School and Hospital of Stomatology, China Medical University | *P. gingivalis* W83 | Overexpressed PG1424(PPAD) gene with His-Tag |
| *P. endodontalis*  ATCC 35406 | American Type Culture Collection (ATCC, Manassas, VA, USA) |  |  |
